# Global transcriptome analysis of the maize (*Zea mays* L.) inbred line 08LF during leaf senescence initiated by pollination-prevention

**DOI:** 10.1371/journal.pone.0185838

**Published:** 2017-10-03

**Authors:** Liancheng Wu, Mingna Li, Lei Tian, Shunxi Wang, Liuji Wu, Lixia Ku, Jun Zhang, Xiaoheng Song, Haiping Liu, Yanhui Chen

**Affiliations:** 1 College of Agronomy, Synergetic Innovation Centre of Henan Grain Crops and National Key Laboratory of Wheat and Maize Crop Science, Henan Agricultural University, Zhengzhou, China; 2 3Cereal Institute, Henan Academy of Agricultural Sciences/Henan Provincial Key Laboratory of Maize Biology, Zhengzhou, China; 3 Department of Biological Sciences, Michigan Technological University, Houghton, Michigan, United States of America; Estacion Experimental del Zaidin, SPAIN

## Abstract

In maize (*Zea mays*), leaf senescence acts as a nutrient recycling process involved in proteins, lipids, and nucleic acids degradation and transport to the developing sink. However, the molecular mechanisms of pre-maturation associated with pollination-prevention remain unclear in maize. To explore global gene expression changes during the onset and progression of senescence in maize, the inbred line 08LF, with severe early senescence caused by pollination prevention, was selected. Phenotypic observation showed that the onset of leaf senescence of 08LF plants occurred approximately 14 days after silking (DAS) by pollination prevention. Transcriptional profiling analysis of the leaf at six developmental stages during induced senescence revealed that a total of 5,432 differentially expressed genes (DEGs) were identified, including 2314 up-regulated genes and 1925 down-regulated genes. Functional annotation showed that the up-regulated genes were mainly enriched in multi-organism process and nitrogen compound transport, whereas down-regulated genes were involved in photosynthesis. Expression patterns and pathway enrichment analyses of early-senescence related genes indicated that these DEGs are involved in complex regulatory networks, especially in the jasmonic acid pathway. In addition, transcription factors from several families were detected, particularly the CO-like, NAC, ERF, GRAS, WRKY and ZF-HD families, suggesting that these transcription factors might play important roles in driving leaf senescence in maize as a result of pollination-prevention.

## Introduction

Maize (*Zea mays* L.), similar to other flowering plants, undergoes a series of distinct growth phases starting from germination, juvenile and adult vegetative phases, flowering, and reproduction, and ultimately to senescence [1]. Senescence is a major concern in the field of agriculture [2], as it often invariably occurs based on the effects of external stressors and internal factors, including metabolic changes, hormonal levels, and environmental stimuli [3–5]. Leaf senescence involves changes in leaf coloration and is considered as a distinct visual phenomenon of plant life cycles [6]. Leaf senescence comprises various physiological processes, including chlorophyll breakdown, termination of photosynthesis, degradation of proteins and nucleic acids, transport of catabolites and nutrients, and responses to cell death [6–8]. Over the last decade, rapid chlorophyll degradation has been identified as one of the earliest events initiated in the chloroplast during green organ senescence [9,10], and the resulting green-bleaching phenotype is often considered as the visual marker of senescence and maturation [11,12]. Although the major Chl catabolic genes (NYC1, NOL, HCAR, PPH, PAO, RCCR and NYEs/SGRs) have been identified in *Arabidopsis* [12–14], their functional regulation has not been thoroughly explored in maize.

In recent years, several senescence-associated genes (*SAGs*) have been identified in various species at the transcriptional level [5,8,15–17]. Early senescence has been induced in the inbred maize line B73 by preventing pollination [18]. Furthermore, with advancements in genome sequencing and global gene expression profiling tools, several studies have evaluated global transcriptomic reprogramming during natural and induced senescence [15,19,20]. Transcriptional and metabolic changes associated with early senescence were evaluated in B73 by microarray analysis [21], which indicated that the sugars play a major role in senescence initiated by pollination prevention. Gene Ontology (GO) analysis together with expressional profiling of senescence-related genes from different species has been conducted. In *Arabidopsis*, SAGs identified by various microarray studies are enriched in metabolic processes, carbohydrate synthesis and photosynthesis [15,22]. However, only a few senescence-initiation genes (SIGs) have been reported in maize, except for some putative senescence regulatory genes (i.e., *ZmNYE1*, *ZmORE1*, *ZmWRKY53*, and *ZmPIFs*) [23]. Furthermore, phytohormones, including abscisic acid, ethylene, auxin, jasmonic acid and cytokinin, have been extensively studied as these play various roles in a wide range of biological processes in plants [6,24–27]. Nevertheless, in maize, most of the underlying mechanisms of leaf senescence remain unclear, and a better understanding of the leaf senescence process in this species is really imperative.

In this study, the maize inbred line 08LF was derived from American germplasm with a good lodging-resistance, high yield, high grain dehydration rate and mechanical harvesting suitability, as well as more prominent premature-senescence and breeding significance. The maize inbred line 08LF was used in this study as the target model plant because of its distinct early-senescence phenotype during pollination-prevention. By analyzing changes in chlorophyll content and identifying DEGs using RNA-Seq and bioinformatics analysis, a total of 5,432 DEGs were identified. Pathway enrichment of DEGs in each expression pattern revealed a complex regulatory network, including various senescence-initiation genes, specific transcript factors, and hormones, which provides an enhanced understanding of the complex molecular processes associated with the onset of the leaf senescence in maize during pollination-prevention.

## Materials and methods

### Plant materials, growing conditions, and sampling details

To identify the genes involved in early leaf senescence, the maize inbred line 08LF, which is known to undergo early leaf senescence when prevented from pollinating during maize breeding, was used in this study. In the summer of 2014, 08LF plants were planted at the farms of Henan Agricultural University (Zhengzhou, China; E113°42', N34°48'), where the average temperature is 14.3°C and the average rainfall is 640.9 mm per year. The 08LF line was manually planted in rows 4-m-long row with a plant spacing of 66 and 30 cm for each field plot. Three fields plot were designed with 20 rows for further investigation (S1 Fig). The primary as well as subsequent ears of the experimental plants were protected with shoot bags prior to silk emergence and were checked every day to record the silking date. Six days after silking (DAS), the shoot bags were removed, and the control plants were allowed to undergo open pollination. The ear leaves of three biological replicates of pollinatied and non-pollinated plants were collected at 9:00 am at 6, 10, 14, 18, 21, and 24 DAS. Then, the middle region of the ear leaves was carefully collected by cutting with a knife, rapidly frozen in liquid nitrogen, and then stored in a -80°C freezer. To verify the stability of the observed phenotype, the chlorophyll content of the middle region of ear leaves from Xingyang (China; E113°35', N34°79') and Sanya (China; E109°35', N18°29’) was measured in 2013, and those from Zhengzhou in 2014. Two-way ANOVA followed by a *post-hoc* test was performed using SPASS software to explore the effect of the environment.

### Chlorophyll content and photosynthetic maximum quantum yield measurement

To assess dynamic alterations in morphology, we measured the chlorophyll concentration of the collected ear leaves using a soil plant analysis development (SPAD) meter (SPAD-502Plus, Konica Minolta, Tokyo, Japan) and a Dual-PAM 100 chlorophyll fluorimeter according to Gnanasekaran *et al* [28]. SPAD values and the photosynthetic maximum quantum yield (Fv/Fm) of the ear leaves from three biological replicates of 08LF pollinated (FP) and 08LF no-pollinated plants (FNP) were measured at 8:30 am at 6, 10, 14, 18, 21, 24, and 27 DAS. SPAD readings were collected from three ear leaf regions: (a) The top part of the leaf, (b) the middle part of the leaf, and (c) 10-cm away from the leaf base. By contrast, Fv/Fm values were only collected in the middle part of the leaves. Ten plants per plot were examined as the one biological replicate, and the average values were calculated from three biological replicates.

### Determination of plant hormone

A second batch of samples consisting of five plants per replicate was collected as described for RNA-Seq analysis during the leaf senescence process, using three biological replicates for each stage (6, 10, 14, 18, and 21 DAS), and stored at -80°C until further analysis for the respective phytohormones JA. The endogenous JA levels of ear leaves and kernels were measured using the enzyme-linked immunosorbent assay (icELISA) described by Wang et al.[8] and Ouyang et al.[29]. The concentrations of phytohormone were then calculated according to Weiler et al. [30].

### Transcriptomic sequencing

RNA was extracted from the middle part of the leaves located near the ear using an RNA Prepure Plant Kit (TIANGEN, Beijing, China). At each time point (6, 10, 14, 18, 21, and 24 DAS for the PNP plants and 10, and 24 DAS for the FP plants), five plants were mixed for each biological replicate, and three biological replicates were used for RNA-Seq analysis. Libraries of each RNA sample were constructed using Illumina Truseq RNA Sample Prep kits (Illumina, Santiago, CA, USA). Single-end sequencing was performed in an Illumina HiSeq2000 sequencer. The entire original sequence data in fastq format were deposited in the NCBI Short Read Archive under Accession Numbers PRJNA347500 and SRP091292.

### Identification and functional analysis of DEGs

Principal component analysis (PCA) was performed using the expression data of genes identified by RNA-Seq at different developmental periods of FP and FNP plants and using OmicShare tools, a free online platform for data analysis (www.omicshare.com/tools), using default parameters. Variables (each sample at each time point for both genotypes) were centered and then normalized. The generated data were then compressed to produce two novel independent variables, which were respectively designated as principal components (PC1 and PC2) and determined to be orthogonal to each other.

An in-house Perl script was employed to remove paired-end reads that consisted of >5% ambiguous residues (Ns), in addition to those showing >10% bases and with a Phred quality score of <20 [17]. DEGs identification was performed as described elsewhere [31]. The remaining high-quality reads from each sample were then mapped to the maize cv. B73 RefGen_V3 genomic DNA sequence (http://www.maizegdb.org) using Tophat software (v2.0.6). Cuffdiff (v2.0.1) was then run using default parameters to estimate changes in expression as well as the associated q-values (the p-values were adjusted using a false discovery rate) of each gene [32]. Finally, the genes assigned significance in the result files were identified as DEGs.

Functional analysis was performed using Gene Ontology (http://www.geneontology.org/) and WEGO (http://wego.genomics.org.cn/). The Cytoscape (v3.0.2) plugin ClueGO + Cluepedia v2.1.3 [33,34] was employed for gene function enrichment analysis [35] of the identified DEGs in the FNP plants, specific up- and down-regulated genes in FNP plants. GO term fusion and restriction with *P* < 0.05 were employed, which integrate GO categories and create a functionally organized GO category network based on overlap between different GO categories and significance. The intervals of each GO level were set at a min level = 5 and a max level = 11, and a 2 gene minimum per category as described by Ku et al.[36] Analysis was conducted using a right-sided hypergeometric test for enrichment based on the ClueGO *Zea Mays* L. reference genome.

### Clustering of DEG expression patterns and transcription factors (TF) enrichment analysis

All these DEGs were functionally annotated and classified into hierarchical categories using the MapMan functional classification system [37]; then, significantly over-represented functional categories were identified based on Fisher’s exact test according to a previous publish [38]. In order to define the dynamic patterns of DEGs expression with leaf development, SOTA (self-organization tree algorithm) clustering based on Pearson’s correlation in the MEV program [39] was used to group DEGs. Clusters in heat-maps were generated by arbitrarily setting a distance threshold (hierarchical clustering) by using MEV program. Transcription factor annotation for the maize genome was acquired from the plant transcription factor database (PlantTFDB v2.0) [40]. A total of 334 TFs belonging to 43 families were analyzed. The numbers of TFs in each comparison were recorded for Fisher’s Exact Test (Fisher test function in the R package). The numbers of TFs in each family in the maize genome were used as background values. The *p* values were corrected for the number of clusters tested using FDR. Corrected *p* values <0.05 were considered significantly enriched.

### Quantitative real-time PCR (qPCR) validation

Total RNA was extracted from FP and FNP ear leaves and grains using TRIzol (Invitrogen, Carlsbad, CA, USA), following the manufacturer's instructions. Samples from three biological replicates were collected. The integrity of RNA samples was verified using 1% agarose gel electrophoresis. Reverse transcription (RT) reactions using the RNA that was extracted from the three independent biological samples was then performed using HiScript Q RT SuperMix for qPCR (Vazyme, China) with a final volume of 20 μL, according to the manufacturer's instructions. Real-time PCR was performed by using a LightCycler® 480II Real-Time PCR detection system (Roche) with SYBR Green Ⅰ. Each reaction included 0.5 μL of the RT reaction product, 0.6 μL of each primer (forward and reverse), 1 μL of 20× SYBR Green Ⅰ dye, 12.5 μL of a premix (BioTeke, China), and 9.8 μL of nuclease-free water. All qRT-PCR reactions were performed in a 96-well plate at 95°C for 3 min, followed by 40 cycles under the following conditions: 95°C for 10 s, 60°C for 20 s, and a final extension at 72°C for 20 s. All reactions were performed in triplicate. The *18S* gene was used as the endogenous control. The specificity of each primer pair was verified by agarose gel electrophoresis and melting curve analysis. The relative abundance of each cDNA was calculated by a comparative CT method (-ΔΔCT) using the formula 2^−ΔΔCT^ [41].

## Results

### Characterization of leaf senescence in inbred line 08LF

**Fig 1:**
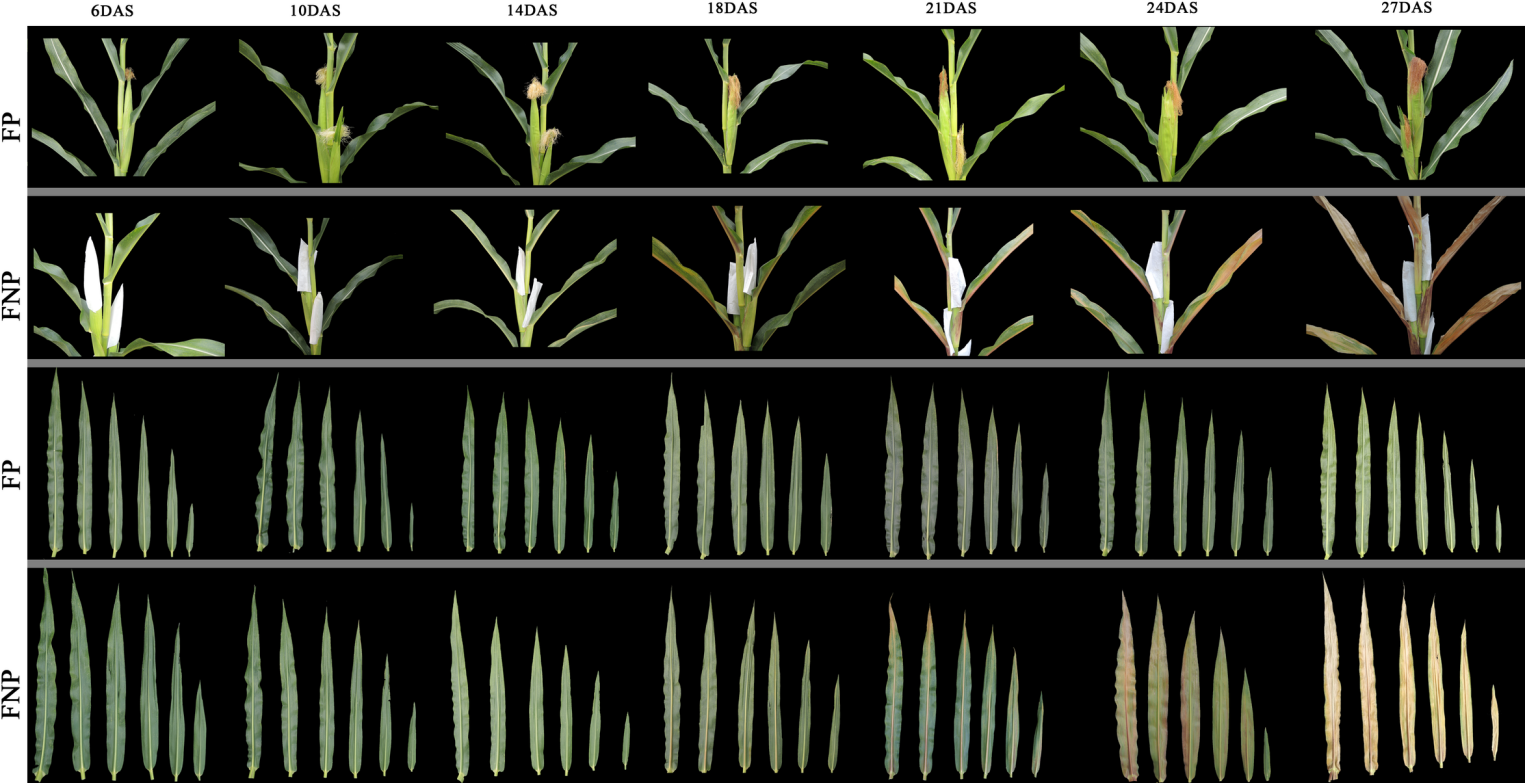
Phenotypes of maize ear leaves and upper leaves during leaf senescence development after silking. Each sample was randomly selected from uniform plants.

**Fig 2:**
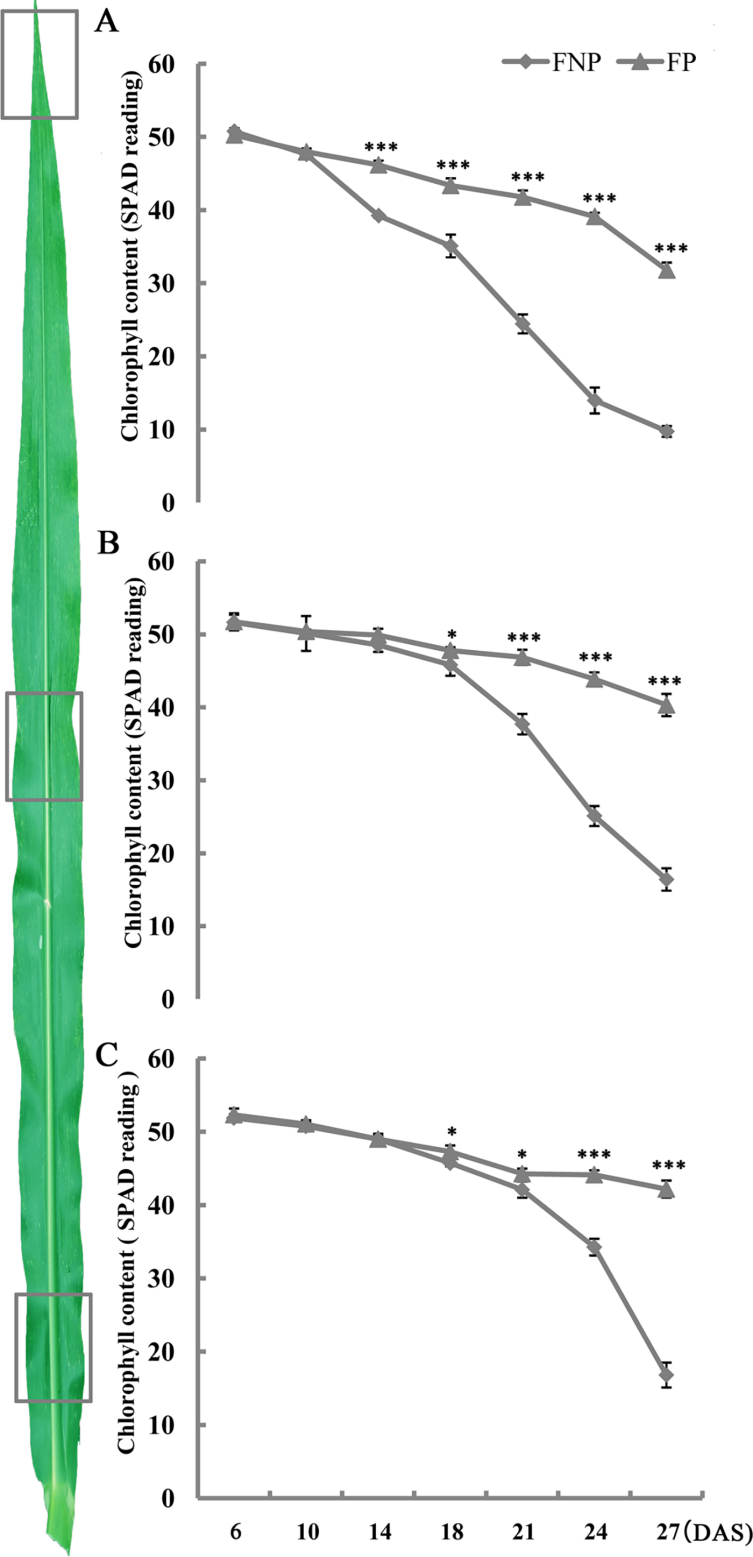
Chlorophyll contents in the leaves of FNP and FP plants during senescence. (A-C) Chlorophyll levels decreased over time in both treatments at different rates. The data were derived from three biological replicates; five plants were mixed to form one biological replicate, and the standard deviation is plotted. The bar represents the mean ± SE, n ≥ 8. *, *p* < 0.05; **, *p* < 0.01; and ***, *p* < 0.001.

To characterize the growth of 08LF, we compared the phenotypes of 08LF, Zong 3, 87–1, Mo17, PH4CV, PH6WC, Chang 7–2, Zheng 58, HCL645 and B73 under pollination prevention. 08LF showed earlier and faster leaf senescence than the HCL645 line and B73 lines, whereas the other inbred lines remained green in color (S2A and S2B Fig). For further investigation of premature leaf senescence in the inbred line 08LF, phenotypes of the ears leaves and the upper leaves of FP and FNP plants were observed in the field at 6, 10, 14, 18, 21, 24, and 27 DAS. Two-way ANOVA followed by a *post-hoc* test was performed to explore the effect of the surrounding environment by measuring the chlorophyll content of the middle region of ear leaves from Xingyang and Sanya in 2013, and Zhengzhou in 2014 (S1 Table). The result showed that the variation between these environments was not significant (*p* > 0.05), which indicated that the phenotypes observed in the same period were not affected by the environment (S1A and S1B Table). From 6 to 21 DAS, the leaves of FP plants remained green. However, the leaf veins of the FNP plants showed a slight red coloration starting at 18 DAS, and the top region of the leaves lost its green color at 21 DAS, whereas the leaves of the FP plants showed a slight green color at 24 DAS. From 27 DAS, the FNP plants showed signs of wilting, whereas all the leaves of the FP plants remained green (Fig 1). In addition, more extensive yellowing of the top section of the leaves from FNP plants was observed from 14 DAS (Fig 2A), in the middle part from 18 DAS (Fig 2B), and in the basal region from 21 DAS (Fig 2C). Degradation of chlorophyll is one of the earliest events of induced senescence [21]. However, the FP plants only showed a relatively slight reduction in the chlorophyll content at the same stages. These results indicated that leaf senescence in the FNP plants occurred earlier than that in the FP plants, and the samples collected at various time points probably reflect the progression of leaf senescence and can be used for the identification of SIGs.

### Identification of DEGs at various time points using RNA-Seq analysis

To examine the transcriptomic changes associated with induced senescence, eight libraries were sequenced using the Illumina deep-sequencing technique, representing time points of 10 and 24 DAS for the FP plants and 6, 10, 14, 18, 21, and 24 DAS for the FNP plants. Three biological replicates for each time point were used for the analysis. A total of 8~10 million raw reads were obtained from each library (SRA submission numbers: PRJNA347500 and SRP091292). After filtering out the low-quantity reads, clean reads (more than 91%) were generated for each sample. The clean reads were then aligned to the maize B73 genome (RefGen_V3); approximately 80% of these are unique reads that can be mapped (Table 1).

**Table 1:** Overview of the sequencing reads obtained from each sample.

Sample	rawdata_count	count_ after_qfilter	reads_keep%	mapped_reads	mapped%	Unique_ mappled_reads	Unique_ mappled_reads%
6DASN_r1	7975845	7834130	98.22	7143579	91.19	5649510	79.09
6DASN_r2	9193409	9019453	98.11	8246352	91.43	6726028	81.56
6DASN_r3	7758852	7591747	97.85	6973570	91.86	5744227	82.37
10DASN_r1	7442024	7291875	97.98	6731287	92.31	5480898	81.42
10DASN_r2	9376032	9181843	97.93	8479906	92.36	7106564	83.80
14DASN_r1	7309638	7172950	98.13	6539700	91.17	5201404	79.54
14DASN_r2	9131516	8960615	98.13	8152781	90.98	6614386	81.13
14DASN_r3	8093502	7922126	97.88	7209648	91.01	5824748	80.79
18DASN_r1	8385772	8229490	98.14	7541812	91.64	6252230	82.90
18DASN_r2	8806960	8617639	97.85	7918925	91.89	6604132	83.40
18DASN_r3	7917699	7740348	97.76	7123355	92.03	5895384	82.76
21DASN_r1	7590268	7437514	97.99	6794370	91.35	5563658	81.89
21DASN_r2	7860744	7698098	97.93	7018891	91.18	5801329	82.65
21DASN_r3	11024139	10794584	97.92	9832230	91.08	8365402	85.08
24DASN_r1	9382351	9163732	97.67	8367413	91.31	7079381	84.61
24DASN_r2	8201533	8054678	98.21	7433771	92.29	6190232	83.27
10DAS_r1	7941949	7777455	97.93	7134198	91.73	5833995	81.78
10DAS_r2	8834814	8644115	97.84	7960889	92.10	6607957	83.01
24DAS_r1	8599542	8432296	98.06	7728019	91.65	6398312	82.79
24DAS_r2	9545411	9339804	97.85	8557539	91.62	7181523	83.92
24DAS_r1	8206919	8027609	97.82	7381500	91.95	6095486	82.58

DAS, indicateds the number of days after silking that the pollinated plants were collected; DASN, indicates the number of days after the no-pollinated plants were harvested; The replicates are defined as r1, r2, and r3, respectively.

PCA of various biological replicates indicated that the replicates and the sequencing platform were highly reproducible (S3A Fig). In addition, the results of PCA analysis results also indicated that the transcriptomic pattern of the leaves from both FP and FNP plants was apparently similar at 14 DAS, and subsequently diverged at 18 DAS. These observations are coincident with the occurrence of senescence in leaves at 18 DAS (Fig 1).

**Fig 3:**
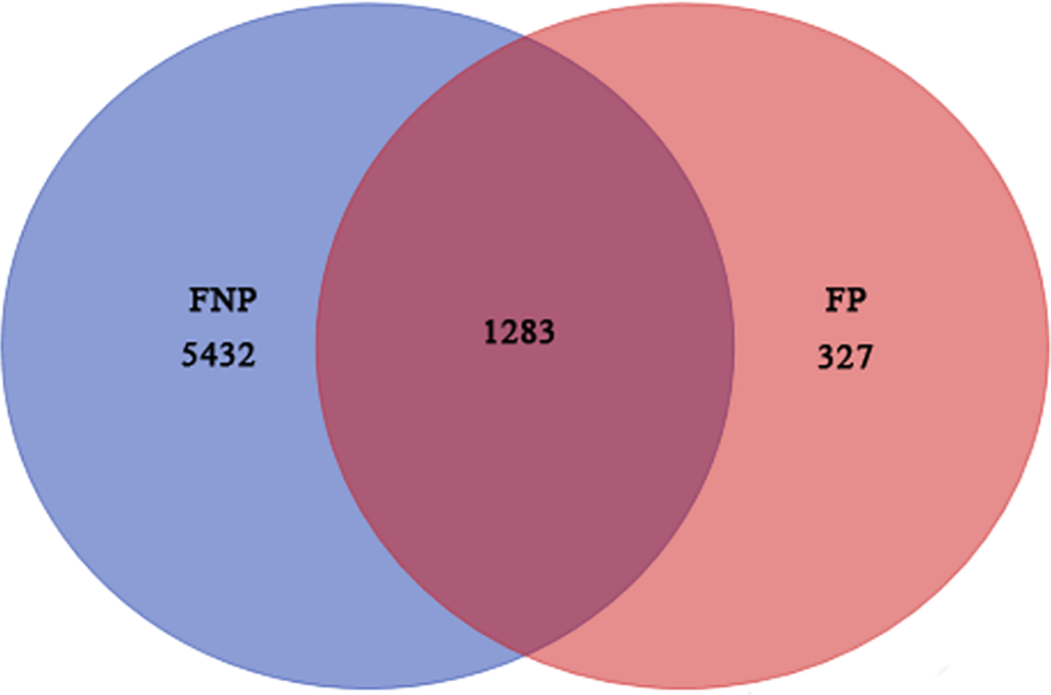
Venn diagram of DEGs identified in FNP and FP plants. The differentially expressed genes shared between FNP and FP plants. Red numbers indicate the gene identified in FP from 6 to 24 DAS; numbers of differentially expressed genes in FNP plants from 6 to 24 DAS are shown in blue.

Putative DEGs were selected based on the following two parameters: a) the average fold-change (> 2) in at least one-time point and b) the FDR was < 0.001. Accordingly, a total of 6,715 and 1,610 DEGs were identified in FNP and FP plants, respectively. Fig 3 shows that approximately 1,283 DEGs genes were shared by these two materials. The present study focused on DEGs associated with non-pollination-related senescence; therefore, 5,432 DEGs were used in the subsequent identification of genes involved in leaf senescence. Among these, 1,925 DEGs were detected to be down-regulated and 2,314 were upregulated in the FNP plants (S3B Fig). These results indicated that the inbred line 08LF exhibited normal senescence, and may present a relatively different regulatory mechanism when prevented from pollinating.

### Functional classification of DEGs

**Fig 4:**
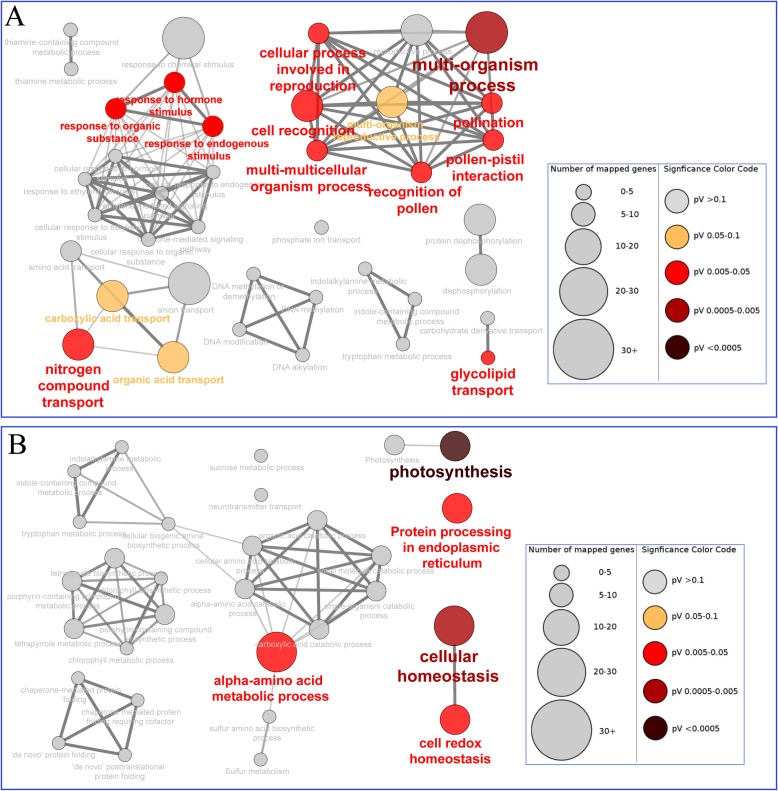
GO enrichment analysis involved in biological processes. Specific up- and downregulated DEGs of FNP plants were analyzed using the Cytoscape plug-in ClueGo + Cluepedia to detect statistically enriched GO categories relative to the ClueGO maize reference genome involved in biological processes (BP). (a) ClueGO plot in GO_BP for specific upregulated genes. (b) ClueGO plot in GO_BP for specific down-regulated genes. Nodes indicate a specific GO term and are clustered based on similarities in their associated proteins. Each node relates to a single GO term and is represented by a specific color according to enrichment significance (pV = p value). Node size represents the number of proteins that were mapped to each category. Edge thickness indicates the calculated kappa score, which is based on the number of proteins that are shared among various terms. Functional groups are indicated by the most significant term relating to the group. Arrows represent positive regulation.

To facilitate better understanding of the biological networks of the specific up- and down regulated DEGs, the functional enrichments were performed using the Cytoscape plug-in Cluego + Cluepedia based on biological processes as described by Wu et al.[42]. As shown in Fig 4A, the specific upregulated genes in the FNP plants were enriched in various processes, such as multi-organism process, nitrogen compound transport, response to endogenous stimulus, and glycolipid transport. However, the specific downregulated genes were enriched in photosynthesis, cellular homeostasis, protein processing in the endoplasmic reticulum and the alpha-amino acid metabolic process (Fig 4B). These results indicate that either up- or down-regulated DEGs identified in the FNP plants are present in different functional pathways involved in regulating leaf senescence by pollination preventing.

### Leaf senescence dynamics of FNP plants at various time points under pollination-prevention

**Fig 5:**
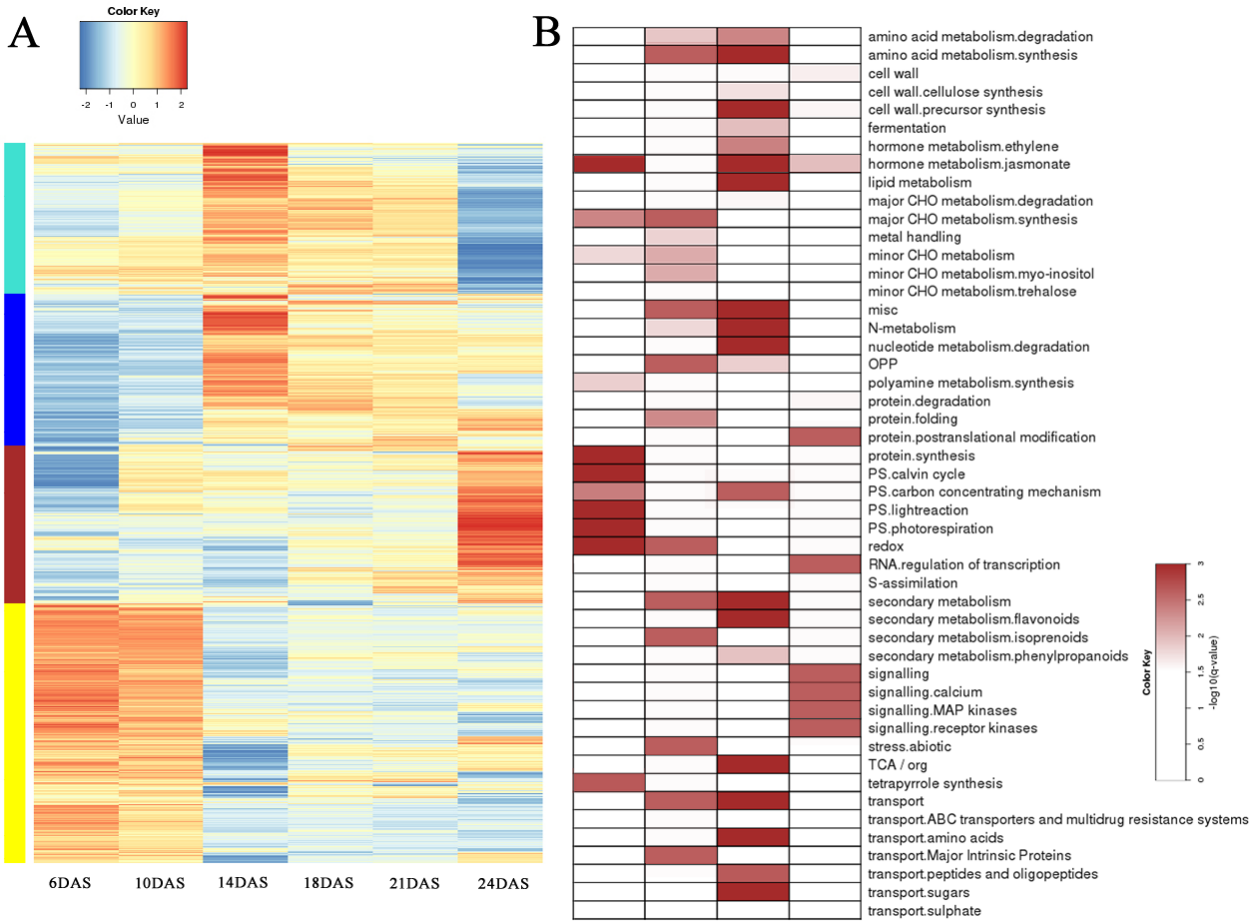
Dynamic transcriptome of the FNP plants during the senescence process. (A) High expression is indicated by the red color, and low expression is showed in blue. Clusters 1 (C1), 2 (C2), 3 (C3), and 4 (C4) are indicated by green, blue, red and yellow, respectively, on the left side. (B) Significant pathways are represented with red color; *p* value < 0.05. C1, C2, C3 and C4 are shown from left to right side.

The DEGs in leaves of the FNP plants at various time points were identified using Pearson’s correlation, which generated four general temporal gene expression patterns during senescence (Fig 5A), and MapMan annotation was used to assign genes into functional categories for each cluster to identify the pathway divergence during the leaf senescence process. As shown in Fig 5, the genes in the C1 cluster had peak expression around 14DAS, after which leaf yellowing was more extensively observed in the top section of the leaves from FNP. However, the expression of these genes in the C1 cluster consistently decreased in their expression until 24 DAS. The most significantly enriched functional categories in this cluster were the jasmonate mechanism, protein synthesis, redox, the Calvin cycle, light reactions and photorespiration of photosynthesis (Fig 5B). The C2 cluster had a similar expression pattern as C1; however, the genes in this cluster showed higher expression at later period of maturity (24 DAS). The enriched categories in this cluster are involved in redox, abiotic stress, transport, secondary metabolism, the synthesis of amino acids metabolism and major CHO metabolism. The expression of genes in the C3 cluster gradually increased until 24 DAS, which was predicated to be associated with the mechanism of early senescence, since the enriched categories in this cluster included hormone metabolism-related genes, such as ethylene and JA metabolism, followed by abiotic stress, protein degradation, TCA, and secondary metabolism of isoprenoids. In addition, the enriched categories in this cluster also included genes involved in transporting sugars, amino acids, peptides and oligopeptides, as well as genes involved in the carbon concentrating mechanism of photosynthesis, misc, N-metabolism, and nucleotide metabolism degradation. The expression of genes in cluster C4 remained relatively high between 6 and 10 DAS, and then decreased from 14 DAS to 24 DAS; this cluster included enriched genes that play roles in RNA regulation of transcription, posttranslational protein modification and signaling-related genes. Taken together, the DEGs in clusters C1 to C4 clusters revealed that the major biochemical shifts in the leaf senescence process are produced partially by highly dynamic, coordinated and localized transitions in mRNA abundance, and pollination prevention affectes gene expression during leaf development.

### Hormone related transcription factors during leaf senescence under pollination-prevention

**Fig 6:**
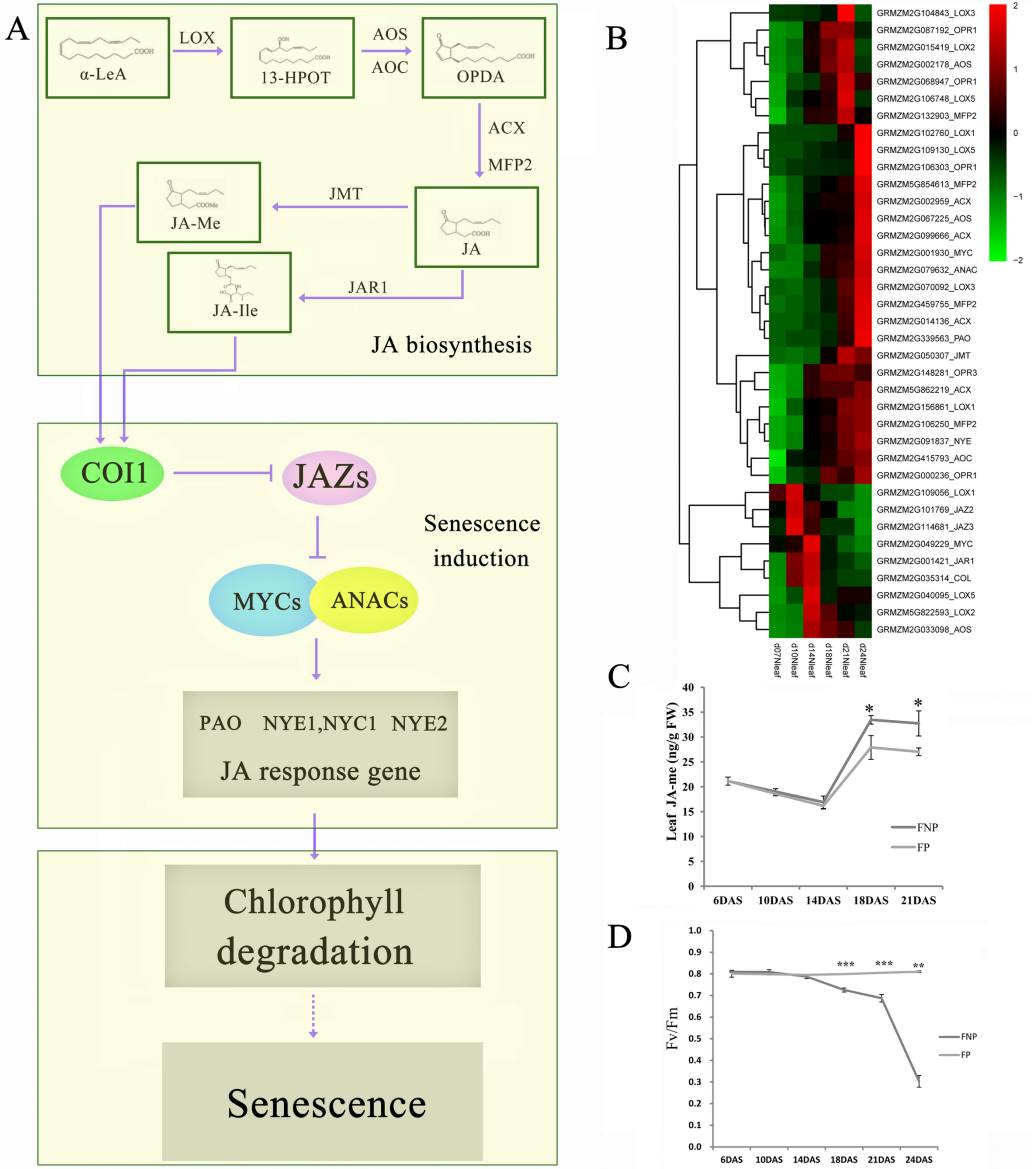
JA-related pathways may play key roles in senescence induction. (A) JA synthesis and signaling pathways with differential expression patterns in FNP plants. (B) Hierarchical clustering of JA-related genes. The color scale indicates the expression value. (C) Leaf JA contents of FNP and FP plants. (D) Changes in the photosynthetic maximum quantum yield (Fv/Fm) in the middle section of ear leaves. The data were derived from five experiments, and the standard deviation has been plotted. The bar represents the mean ± SE, n ≥ 8. *, *p* < 0.05; **, *p* < 0.01; ***, and *p* < 0.001.

The analysis of gene patterns and pathways revealed that DEGs were significantly enriched in photosynthesis mechanisms in the C1 cluster, whereas DEGs were mostly enriched in transcription regulation and hormones (especially JA) from cluster C3. Chlorophylls are essential molecules that harvest solar energy in photosynthetic antenna systems; they are involved in charge separation and electron transport within reaction centers. Both FP and FNP plants exhibited an expected decline in the chlorophyll content with leaf growth (Fig 2). Kyoto Encyclopedia of Genes and Genomes (KEGG) analysis showed that genes involved in JA metabolism were highlighted in the DEG lists (Fig 5B). Eight JA biosynthesis-related genes, encoding ten lipoxygenases (LOXs), one allene-oxide cyclase (AOC), three allene oxidase synthases (AOSs), four acyl-CoA oxidases (ACXs), four enoyl-CoA hydratase/3-hydroxyacyl-CoA dehydrogenases (MFP2s), five 12-oxophytodienoate reductases (OPRs), and one jasmonic acid carboxyl methyltransferase (JMT) were displayed in the DEG list of the FNP plants. In addition, the expression of three genes (JAZ1, JAZ2 and JAZ3) involved in the JA signaling pathway were also significantly differently expressed during leaf senescence process induced by pollination prevention (Fig 6B). Consistently, the JA content in the FNP plants was significantly higher than that in the FP plants at 14 DAS (Fig 6C), whereas the Chl content and photosynthetic maximum quantum yield (Fv/Fm) showed decreased expression patterns in the middle part of leaves from 14 DAS (Figs 2 and 6D). These results indicated that JA-regulated Chl degradation is probably involved in the onset of leaf senescence in FNP maize plants (Fig 6A). In addition, genes related to other hormones were also found in this study, including auxin, ethylene and abscisic acid (S2 Table), which showed that the senescence response caused by pollination-prevention is a complex network involving all kinds of hormones.

As shown in Table 2, a total of 334 TFs from 43 TF families were identified in maize. Six families were statistically overrepresented during maize leaf senescence, including CO-like, ERF, GRAS, NAC, WRKY and ZF-HD. Five of the seven CO-like family genes involved in C1 and C4 were downregulated from 14 DAS, whereas the ERF and NAC families were mainly distributed in C3, and were found to be continually increased throughout all senescence stages. Most DEGs belong to the GRAS and WRKY families, were expressed in C3 and C4 (Table 2 and S3 Table). These results indicated that the complex regulatory mechanism of early senescence in maize are mediated by the TFs under pollination prevention.

**Table 2:** Number of transcription factors (TFs) identified in FNP plants during the senescence process.

Family	C1	C2	C3	C4	Total	TFs in maize
**AP2**	0	1	3	0	4	54
**ARF**	0	1	3	5	9	62
**ARR-B**	1	0	0	0	1	13
**B3**	0	0	0	2	2	77
**bHLH**	2	0	11	8	21	308
**bZIP**	1	1	1	9	12	216
**C2H2**	0	1	2	6	9	179
**C3H**	2	1	0	1	4	110
**CAMTA**	0	0	0	1	1	10
**CO-like**	5^a^	0	0	2^a^	7	18
**DBB**	2	0	0	2	4	20
**Dof**	1	1	0	1	3	52
**E2F/DP**	0	0	0	2	2	24
**EIL**	0	0	0	1	1	9
**ERF**	0	2	20^a^	15	37	204
**FAR1**	0	0	0	2	2	25
**G2-like**	2	1	2	6	11	89
**GATA**	1	2	0	6	9	54
**GRAS**	2	4	9^a^	4^a^	19	104
**GRF**	0	0	0	1	1	32
**HB-other**	0	0	0	1	1	28
**HD-ZIP**	1	2	2	3	8	97
**HSF**	1	4	1	5	11	49
**LBD**	0	0	0	5	5	60
**M-type_MADS**	0	2	0	0	2	46
**MIKC_MADS**	0	0	0	3	3	90
**MYB**	1	4	8	15	13	203
**MYB_related**	6	2	0	9	17	169
**NAC**	0	0	24^a^	11	35	189
**NF-YA**	0	3	0	1	4	36
**NF-YB**	0	1	0	0	1	27
**NF-YC**	2	2	1	1	6	25
**Nin-like**	0	0	1	1	2	23
**RAV**	0	0	0	2	2	3
**S1Fa-like**	0	0	0	0	0	5
**SBP**	0	0	1	1	2	55
**SRS**	0	0	0	1	1	11
**TALE**	0	1	0	1	2	52
**TCP**	0	1	0	2	3	52
**Trihelix**	0	0	3	5	8	57
**WOX**	1	0	0	0	1	30
**WRKY**	3	0	20^a^	20^a^	43	161
**ZF-HD**	3^a^	2	0	0	5	26
**Total**	37	39	112	161	334	3154

^a^ The overrepresented TF families at a *p* value < 0.05 (Fisher’s exact test)

### Validation of DEG expression profiles using RT-qPCR

**Fig 7:**
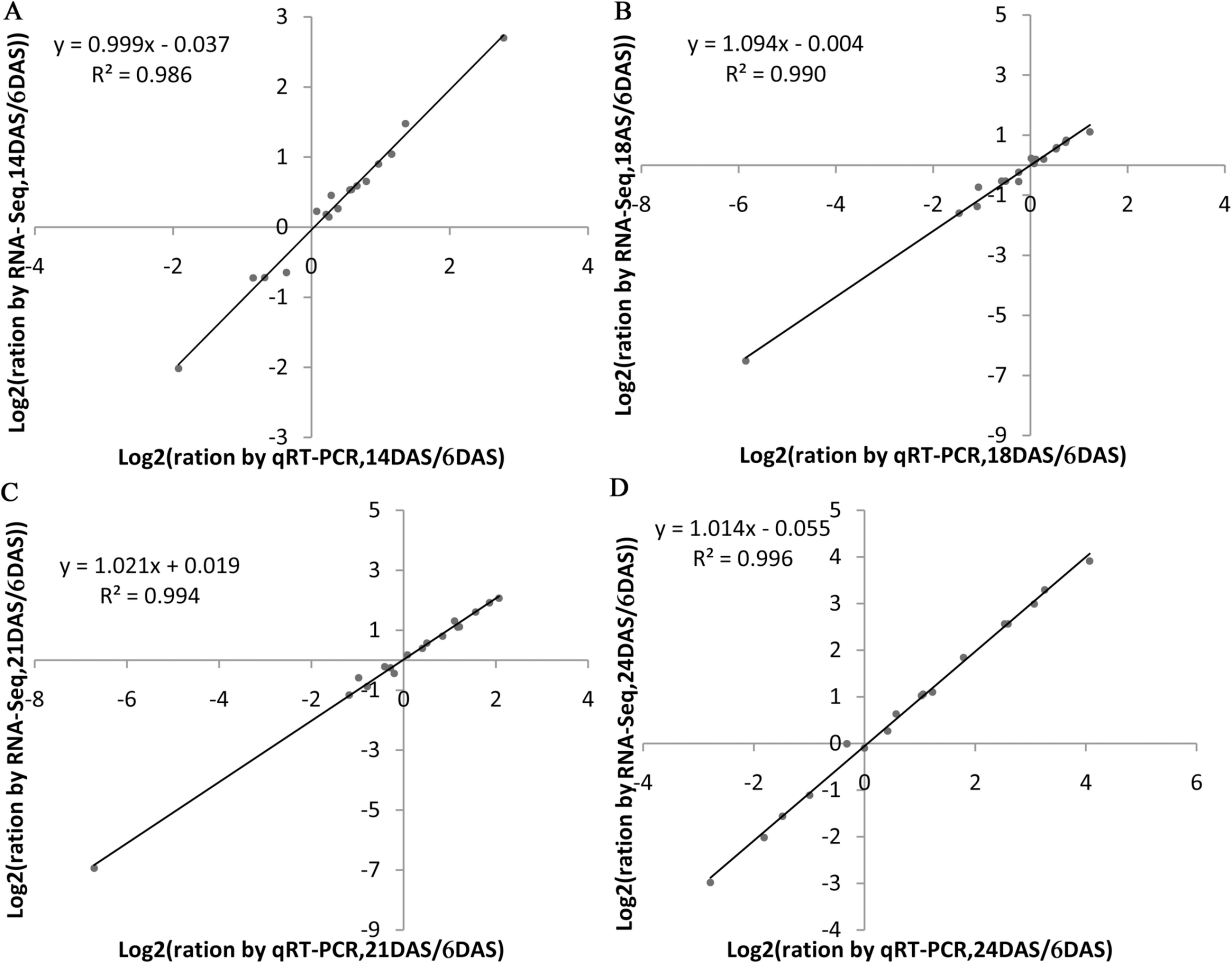
Comparison of the expression ratios of some selected genes using RNA-Seq and qRT-PCR.

To further validate the results of our RNA-Seq data, quantitative RT-PCR analysis was conducted using specific primers based on a subset of 18 DEGs (S4 Table) that were identified by RNA-Seq as being upregulated or downregulated. The results showed that 17 of the DEGs (94%) showed the same expression profile, whereas we could not assess the expression profile of one gene due to a reaction failure, which may possibly be caused by a mutation at the site where our primer annealed. In addition, the expression profiles of the 17 genes were consistent with our RNA-Seq ratios, with a relative R^2^ of >0.980 (Fig 7). Our findings thus indicate that our DEG-based technique for measuring transcript abundance is precise and can be employed in the analysis of gene expression in an organism that has no available genome information.

## Discussion

Senescence, as the final step of plant growth and development, is highly correlated with the crop yield [43]. Early leaf senescence can generally decrease yield but elevate nitrogen use efficiency under conditions of nitrogen starvation. Delaying leaf senescence may lower nitrogen use efficiency, but may improve the final yield. Thus, fine-tuning the onset as well as the process of leaf senescence are two of the most critical prerequisites for high crop production. In our study, genome-wide transcriptional profiling of ear leaves was performed by inhibiting pollination in the maize inbred line 08LF. By comparing the transcriptomic changes between FP and FNP plants, we have identified a total of 5,432 DEGs. The different phenotypic characteristics during the senescence process between FP and FNP plants revealed that the onset of senescence may occurs from 14 DAS in FNP plants. In addition, the functions of various *SIGs*, especially transcription factors, and hormones-related genes were reported to play significant roles in the leaf senescence-initiation process at the transcriptional level. The study provides an enhanced understanding of the complex molecular processes associated with the onset of leaf senescence in maize.

### Comparative analysis of senescence-induced genes in maize under pollination prevention relative to other plant species

Maize is a major source of food, feed, and fuel. MAP kinase, homeobox genes, cytokinins, and nitrogen levels have been reported to be involved in leaf senescence in maize [44–48]. In addition, removing the maize ears after flowering has been reported to induce the early senescence of the upper leaves of ears [49]. Recently, Wu et al. utilized the inbred line Yu87-1 to determine the potential roles of miRNAs as well as their target genes in leaf senescence by using small RNA deep sequencing technology [50]. Their study identified a total of 16 candidate miRNAs, which then were used in elucidating the regulatory roles of miRNAs in leaf senescence of maize. However, the molecular mechanism underlying the regulation of senescence induction in maize during pollination prevention remains unclear. A previous report showed that preventing pollination in the inbred line B73, which harbors the reference maize genotype, induces early senescence [18]. Comparison of the expression patterns of genes in plants undergoing induced and natural senescence showed that sugars play an important role in pollination prevention-induced senescence [21]. Transcriptional analyses of natural leaf senescence in the maize inbred line Q319 at three phase (mature leaves, early senescent leaves, and later senescent leaves) were conducted by Zhang et al. [51]; the up-regulated genes at the early senescence stage were involved in aromatic amino acid biosynthetic process and transport, cellular polysaccharide biosynthetic process, and cell wall macromolecule catabolic process. By contrast, at the later senescence stage, up-regulated genes were involved in amino acid metabolism, transport, apoptosis, and response to stimulus. Comparative analysis indicated that among the 5,432 DEGs identified in the present study, 1,938 (35.68%) DEGs overlapped with those detected in two previous studies, and 3,494 (64.32%) were specifically identified in this study (S5 Fig). In our study, the specific up-regulated genes in the FNP plants were enriched in various processes, such as multi-organism process, nitrogen compound transport, response to endogenous stimulus, and glycolipid transport. These results showed that leaf senescence induced by pollination prevention was not only involved in the process of natural senescence, such as transport and response to stimulus, but also implicated in novel functions, such as multi-organism process. In addition, genes involved in photosynthesis were specifically down-regulated, and a decline in photosynthetic activity may trigger senescence [52]. Yoshida et al.[53] hypothesized that metabolites such as carbohydrates and amino acids may play a role in the induction and in the progression of senescence. The identified functional enrichment of DEGs will probably provide new directions in the elucidation of the mechanisms underlying early senescence under pollination-prevention.

Previous studies were conducted to better understand leaf senescence, e.g., identification and characterization of various SAG*s* in addition to senescence-related mutants of different plant species, which include *A*. *thaliana*, *Oryza sativa*, *Medicago truncatula*, *Gossypium hirsutum* L., as well as *Sorghum bicolor* [54–59]. These efforts contributed to the identification of a number of transcription factors (i.e., WRKY, NAC, HOMOBOX, and MYB) [60]. With the development of next-generation high-throughput sequencing, RNA-Seq has been used to investigate the expression of SAG genes in various plants [61–63]. A study of developmental leaf senescence in cotton (*G*. *hirsutum* L.) revealed that SAG*s* were functionally enriched in the processes of auxin metabolism, serine and glycine catabolism, and other carbolic-related activities. In addition, several WRKY-, ERF-, NAC-associated genes were previously identified [64]. A survey of leaf senescence has been conducted for switchgrass (*Panicum virgatum*) to explore its underlying molecular mechanism, which indicated that transport processes and NAC transcription factors are enhanced during nutrient remobilization [65]. A comparison of the results of genomic and RNA-Seq in sorghum (*Sorghum bicolor*) was conducted and identified 176 potential markers for monitoring senescence, thereby providing valuable resources for comparative genomics analyses of leaf senescence [66]; however, the senescence mechanism in maize has not been clearly revealed. In our study, all of these kinds of TFs were identified, and showed strong similarity to those of *Arabidopsis* and rice (S2 Table), which indicated that the relationships between TFs and leaf senescence might be conserved in these species.

### Expression patterns of SIGs and the TFs enriched during senescence induction

Several SAG*s* have been identified in various species at the molecular level; however, no *SIGs* have been reported in maize to date. To identify the *SIGs* under pollination prevention, leaves from six different developmental stages covering the senescence process were collected and analyzed at transcriptional level. It was previously reported that the chlorophyll content, membrane ion leakage, and gene expression can be used as senescence markers [5]. In the present study, the middle region of the leaves started to yellow at around 18 DAS (Fig 1), and the chlorophyll content of leaf tips rapidly decreased from 14 DAS (Fig 2A). These findings, together with those described by Lin et al.[64], indicated that the initiation of leaf senescence in the 08LF inbred line under our described growth conditions occurs around 14 DAS [64].

*NYE1*, *LOX2*, *PHT5*, *FAAH*, *ACS2*, *PAO* in *Arabidopsis* and *SAG12* in *sorghum* were upregulated at the initiation of leaf senescence [67–70]. However, although numerous genes related to the leaf senescence process were described previously for various plant species, the onset of premature-senescence in maize remains unknown, especially that induced by pollination prevention. Consequently, the new findings of regulation patterns and functions of DEGs in our study will provide more evidence to discover new genes related to the onset of early senescence induced by the pollination prevention. WRKY53 and AtWRKY6 were previously reported as senescence-associated factors in *Arabidopsis* [71,72]. WRKY TFs have also been shown to act downstream of the defense signaling pathways of mitogen-activated protein kinases and are involved in various JA- and SA-dependent signaling pathways for cellular defense [73]. MYB transcript factors were reported to be involved in plant growth and development, stress response, and hormone signaling. Previous reports have shown that *mybn* mutants display a delayed-senescence phenotype, whereas the AUX-responsive phenotype is observed in MYBH over-expression lines, which in turn causes premature leaf senescence as well as the upregulation of leaf senescence marker genes [74], thereby suggesting that MYB genes mediate leaf senescence through AUX homeostasis. In *Arabidopsis*, an MYB-related TF, *AtMYBL*, promotes early leaf senescence. In addition, *AtMYB2* inhibits cytokinin-mediated branching to regulate whole-plant senescence during later stages of development [75], and AUX negatively regulates local biosynthesis of cytokinins by controlling the expression of isopentenyl transferase (IPT) genes [76]. In the present study, the MYB family was distributed across all four clusters, whereas the NAC and WRKY TFs were specifically enriched during senescence (Table 2), thereby suggesting that these TFs contribute to leaf senescence. A number of NAC genes in *Arabidopsis* have been reported to be positive regulators of senescence [60]. The abundance of NAC TFs in C3 was significantly higher than that of the other profiles, which indicates that the NAC genes in our study underwent sustained unregulated expression, and ultimately, NAC may induce leaf early senescence. Many of NACs are known to be positive regulators of senescence, which have been reported [77,60,78]. However, Kong et al. [79] showed that although 11 upregulated NAC TFs in the early senescence cotton line were identified, the expression level of GhNAC6 in the early-senescence cotton line decreased from 65 to 95 days after pollination (DAP), and then increased from 95 until 110 DAP. In our study, except for the NAC TFs in C3, the NAC TFs in other clusters support Kong's hypothesis that different NAC TFs perform different functions during leaf senescence. Further research on functional studies of leaf senescence-related candidate NAC genes is thus imperative. The present study will lay the basic foundation for discovering novel *SIGs* and senescence-related regulatory networks by verifying the regulation pattern of the senescence-initiation process in the future.

### Role of JA during senescence onset in FNP plants

JA has been considered as an inducer of senescence in various plant species [80–86]. Exogenous application of JA resulting in senescence has also been demonstrated [80]. Rubisco activase (RCA) plays an important role in JA-induced leaf senescence [84]. In *Arabidopsis*, JA levels were higher in senescing than in non-senescing leaves, and JA biosynthesis-related genes were also activated during senescence [80]. Various senescence-associated genes (SAGs) can also be induced by JA [20,87]. For example, the expression of *SENESCENCE 4* (*SEN4*) and *SAG12* was increased after JA treatment in wild-type plants but was severely reduced in the *coi1-2* mutant [88]. Some WRKY TFs were found to be involved in JA-induced senescence. WRKY70 and WRKY53 function as the node of JA- and SA-mediated signals [89–92], and WRKY57, a repressor of *SEN4* and *SAG12*, functions as a node of convergence for JA- and auxin-mediated signaling pathways during JA-induced leaf senescence [27]. miR319-controlled *TCP4* positively regulates leaf senescence by directly activating the JA biosynthetic gene *LIPOXYGENASE 2* (*LOX2*) [70]. Previously, JA also have been reported to promote leaf degreening in other species [85], mechanisms at the molecular level remains unknown in maize. Rapid Chl degradation is a characteristic event during leaf senescence or maturation; therefore, understanding the Chl degradation mechanism will contribute to the exploration of senescence responses. In our study, some core senescence genes formed a complex network involved in JA biosynthesis and signaling pathways in FNP plants (Fig 6A). The expression of most of JA biosynthesis related genes (*MFP2*: *GRMZM5G854613*, *GRMZM2G459755*, *GRMZM2G106250; AOS*: *GRMZM2G067225; ACX*: *GRMZM2G014136*, *GRMZM2G002959*, *GRMZM5G862219*, *GRMZM2G099666; LOX1*: *GRMZM2G156861*, *GRMZM2G102760; LOX5*: *GRMZM2G109130; LOX3*: *GRMZM2G070092; OPR1*: *GRMZM2G106303*) consistently increased during the senescence process, whereas some of them (*LOX5*:*GRMZM2G040095*, *LOX2*: *GRMZM5G822593; AOS*: *GRMZM2G033098*) showed peak expression around 14 DAS (Fig 6A and 6B, S2 Table). In addition, genes (*COI*, *JAZ*, *MYC*, *ANAC*, *NYE* and *PAO*), that were previously reported in other species as the JA signaling transport genes [12–14], were associated with the Chl degradation. In this study, *MYC(GRMZM2G001930)*, *ANAC(GRMZM2G079632)*, *NYE(GRMZM2G091837)*, and *PAO(GRMZM2G339563)* displayed the highest expression level at 24 DAS, whereas the expression of *JAZ* (*GRMZM2G101769* and *GRMZM2G114681*) decreased continually from 10 DAS. Interestingly, the Chl content and photosynthetic maximum quantum yield (Fv/Fm) were decreased from 14 DAS. The JA level in the FNP plants was much higher than that in the FP plants at 14 DAS (Figs 2, 6C and 6D). This result indicated that JA is probably required at a high concentration for the onset of the senescence process, and an increase in the JA content might positively regulate maize senescence, as suggested for other species, through Chl degradation in ear leaves.

By observing the phenotype of the ears under the pollination-prevention, we found that the development of ears was apparently different under pollination-prevention compared to the natural plants (S5 Fig), and we detected the expression of the JA-related genes and the JA content in the grains (S6 and S7 Figs). Most of the JA biosynthesis genes exhibited higher expression in the FNP than in the FP plants, but the level of *LOX3(GRMZM2G070092 and GRMZM2G104843)* and *JAR1(GRMZM2G001421*) changed. Previously, *ZmLOX3* was shown to serve as a negative regulator of JA biosynthesis in roots and seed. The *lox3* knockout mutant roots produced elevated basal levels of JA and displayed an increased expression of JA biosynthesis genes [93], suggesting that *LOX3* may suppress the production of JA. The result also indicated that JA signaling transport genes showed a different expression level in the maize grains of FP plants, which exhibited a relatively stable change compared with the FNP plants but with opposite expression of *JAZ* genes. In addition, the JA content has been reported to be increased during tomato embryo and seed development [94]. Interestingly, in our study, the content of JA in FNP was higher with a peak at 18 DAS, than that in FP plants, which exhibited a continual increase (S7 Fig). These results revealed a very different expression tendency of the JA-related genes and JA content in grains in comparison with the ear leaves, and JA mediated the plant senescence in FNP plants needs to be further explored.

Previous studies have reported that the initiation and procession of leaf senescence in plants are affected by phytohormones [6–8,26], particularly ABA, which is considered to promote senescence, whereas cytokinin and AUX delay this process. AUX has been reported to be a negative regulator of leaf senescence [7], but an increase in the auxin content has also been observed during leaf senescence [1]. Other hormones, such as GA3 and ZR, have also been reported to be associated with leaf senescence process [80,95,26], but the function of GA3 and ZR in senescence is poorly understood, compared to ABA, JA and cytokinin. In this study, many hormone-related genes were identified; however, these genes showed different expression patterns (S2 Table), which indicates that the regulation of hormone response to the leaf senescence in maize under pollination-prevention is a complex process. In addition, how these hormone-related genes contribute to the onset of senescence in maize by pollination prevention needs to be addressed in future.

Taken together, our results show that FNP plants undergo senescence earlier compared to FP plants. Phenotypic analysis, the chlorophyll content, and RNA-Seq data were used to identify a total of 5,432 DEGs, and GO functions were enriched in the natural senescence-related processes such as transport and response to stimulus, as well as in several novel functions such as multi-organism process and photosynthesis. In addition, the expression patterns of all DEGs were enriched in four clusters. Furthermore, many TFs, and hormone-related genes were found in each cluster, indicating that these associated genes may be the predicated major regulatory cues to induce early senescence under pollination-prevention. Through the analysis of pathway enrichment in each cluster, JA metabolism was predicated as the one of main cues for the senescence initiation response, and the JA content was higher in FNP than that in FP plants from 14 DAS. More importantly, exploration of early-senescence in the inbred line 08LF, will provide a theoretical foundation for maize production in understanding the mechanism of premature senescence.

## Supporting information

S1 Fig Diagrams of samples collection of RNA-Seq on the field. Three biological replicates were independently derived from Sample plot 1, 2, and 3. The sample plots were indicated by green, and the red for hybrid line.
(TIF)

S2 Fig Chlorophyll content of the leaves during senescence by pollination -prevention. (A) Changes in chlorophyll content in 10 elite inbred lines that were prevented from pollinating. (B) Changes in chlorophyll content in 10 inbred lines under natural pollination. The data are derived from five experiments and the standard deviation plotted. The bar represents the mean ± SE, n ≥ 8. *, *p* < 0.05; **, *p* < 0.01; ***, and *p* < 0.001.
(TIF)

S3 Fig Bioinformatics analysis of transcriptome sequence data in maize. (A) Pearson’s correlation coefficient between biological replicates. (B) The regulation pattern in FNP plants during senescence.
(TIF)

S4 Fig The overlap of differentially expressed genes during natural leaf senescence and induced leaf senescence. The green area represents the differentially expressed genes during natural leaf senescence by Zhang et al. (2014), the blue area represents the differentially expressed genes in B73 during early leaf senescence induced by prevent pollination by Sekhon’s et al. (2012), the yellow area represents the differentially expressed genes in this study. The areas shown in the diagram are not proportional to the number of genes in each group.
(TIF)

S5 Fig The phenotypes of maize ear in FNP and FP plants at 7 DAS (left side) and 21 DAS (right side). (TIF)

S6 Fig Expression level of JA-related genes involved in JA synthesis and signaling pathways in the grains of FNP and FP plants. (PDF)

S7 Fig The content of JA-me in the grains of FNP and FP plants. The bar represents the mean ± SE, n ≥ 8.
(TIF)

S1 Table Two-way ANOVA of the effect of the environment on FP and FNP plants. (XLSX)

S2 Table List of JA metabolism-related genes in. Excel file containing a list of alpha-linolenic acid metabolism-related genes in FNP plants.
(XLSX)

S3 Table Differentially expressed TFs in each FNP plant cluster. (XLSX)

S4 Table Primers used for RT-PCR analyses. (XLSX)
