# Supplementary figures and images for: Global transcriptome analysis of the maize (Zea mays L.) inbred line 08LF during leaf senescence initiated by pollination-prevention
Source: PLoS One. 2017 Oct 3;12(10):e0185838. doi: 10.1371/journal.pone.0185838 (PMC5626513; doi:10.1371/journal.pone.0185838)

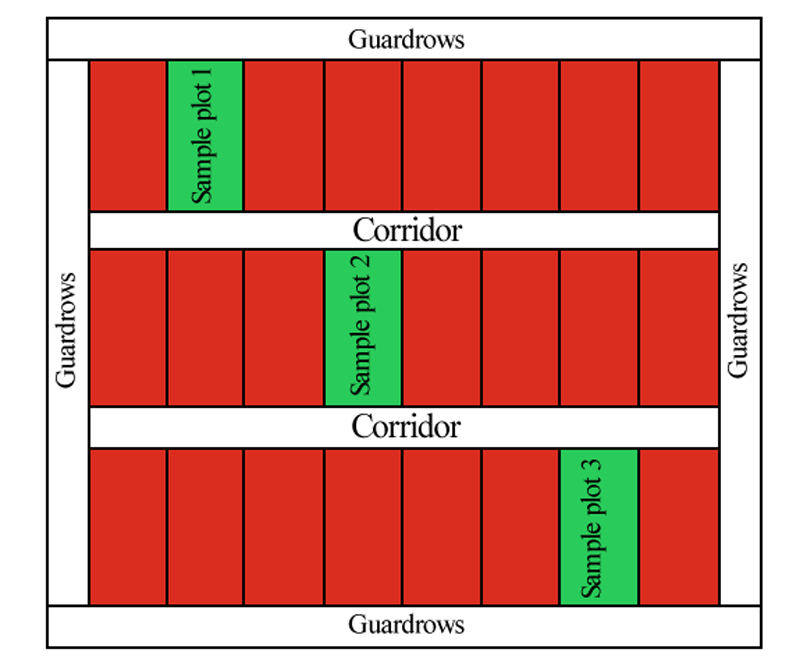

Supplement: S1 Fig — Three biological replicates were independently derived from Sample plot 1, 2, and 3. The sample plots were indicated by green, and the red for hybrid line. (TIF) [file pone.0185838.s001.tif]

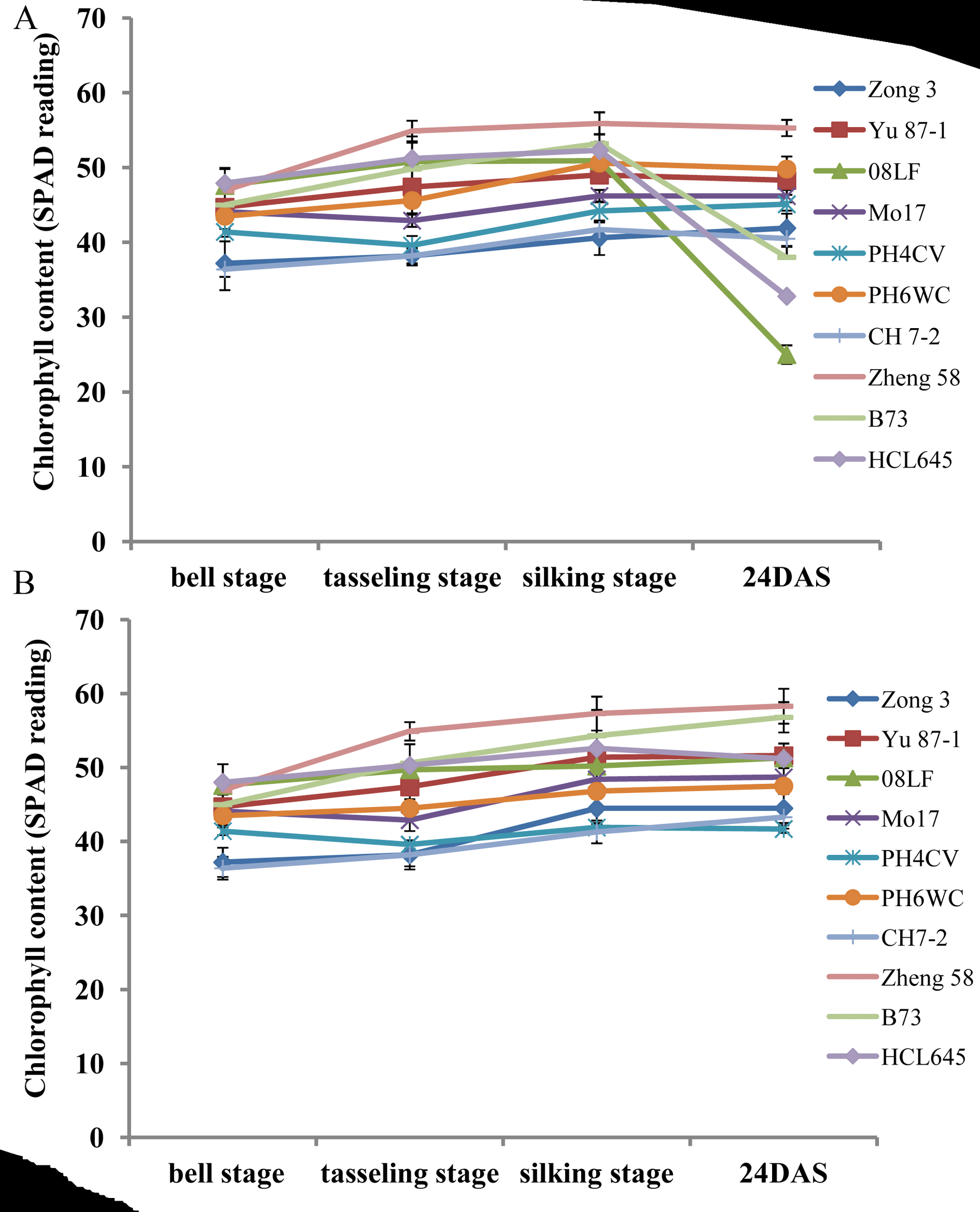

Supplement: S2 Fig — (A) Changes in chlorophyll content in 10 elite inbred lines that were prevented from pollinating. (B) Changes in chlorophyll content in 10 inbred lines under natural pollination. The data are derived from five experiments and the standard deviation plotted. The bar represents the mean ± SE, n ≥ 8. *, p < 0.05; **, p < 0.01; ***, and p < 0.001. (TIF) [file pone.0185838.s002.tif]

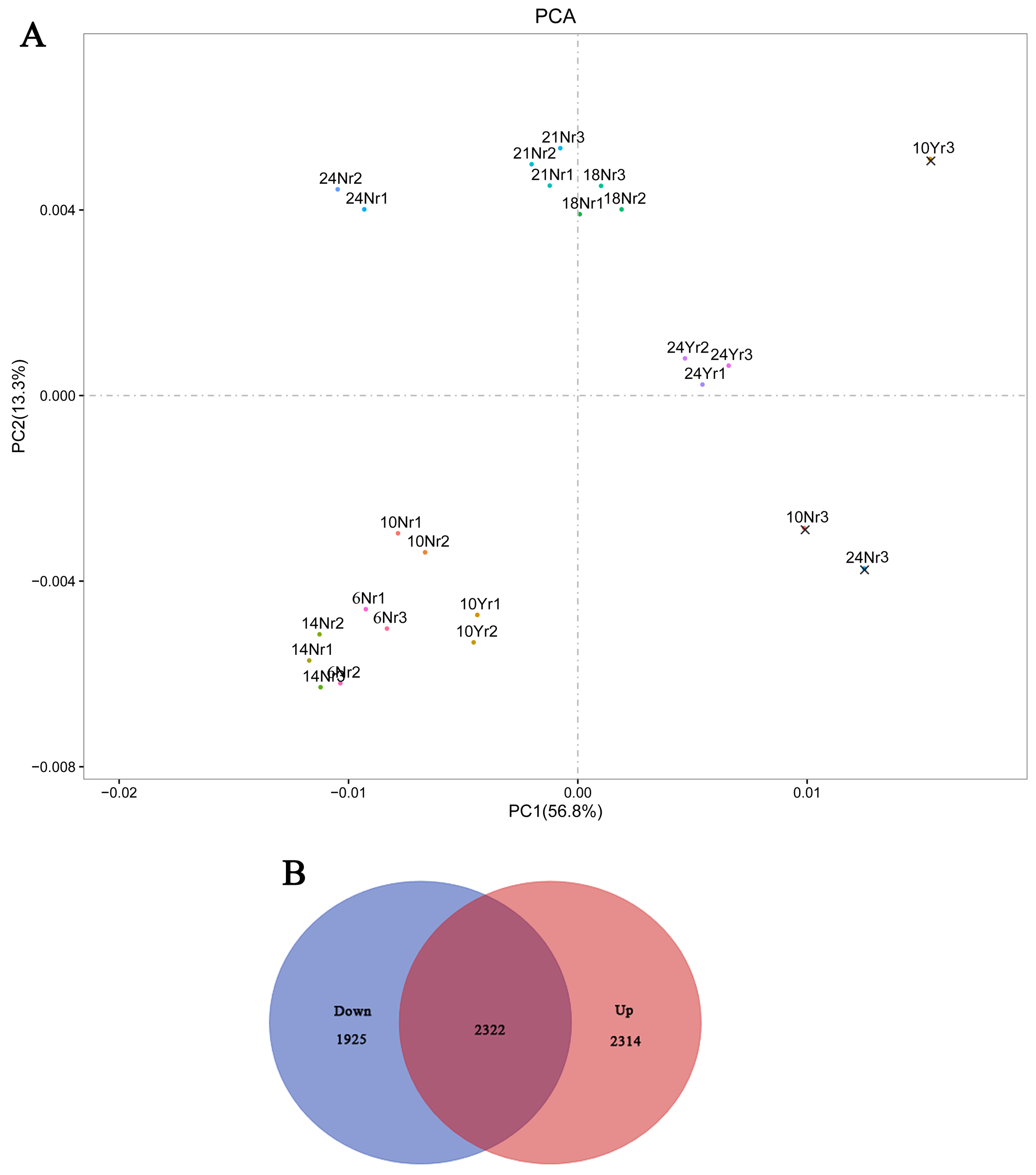

Supplement: S3 Fig — (A) Pearson’s correlation coefficient between biological replicates. (B) The regulation pattern in FNP plants during senescence. (TIF) [file pone.0185838.s003.tif]

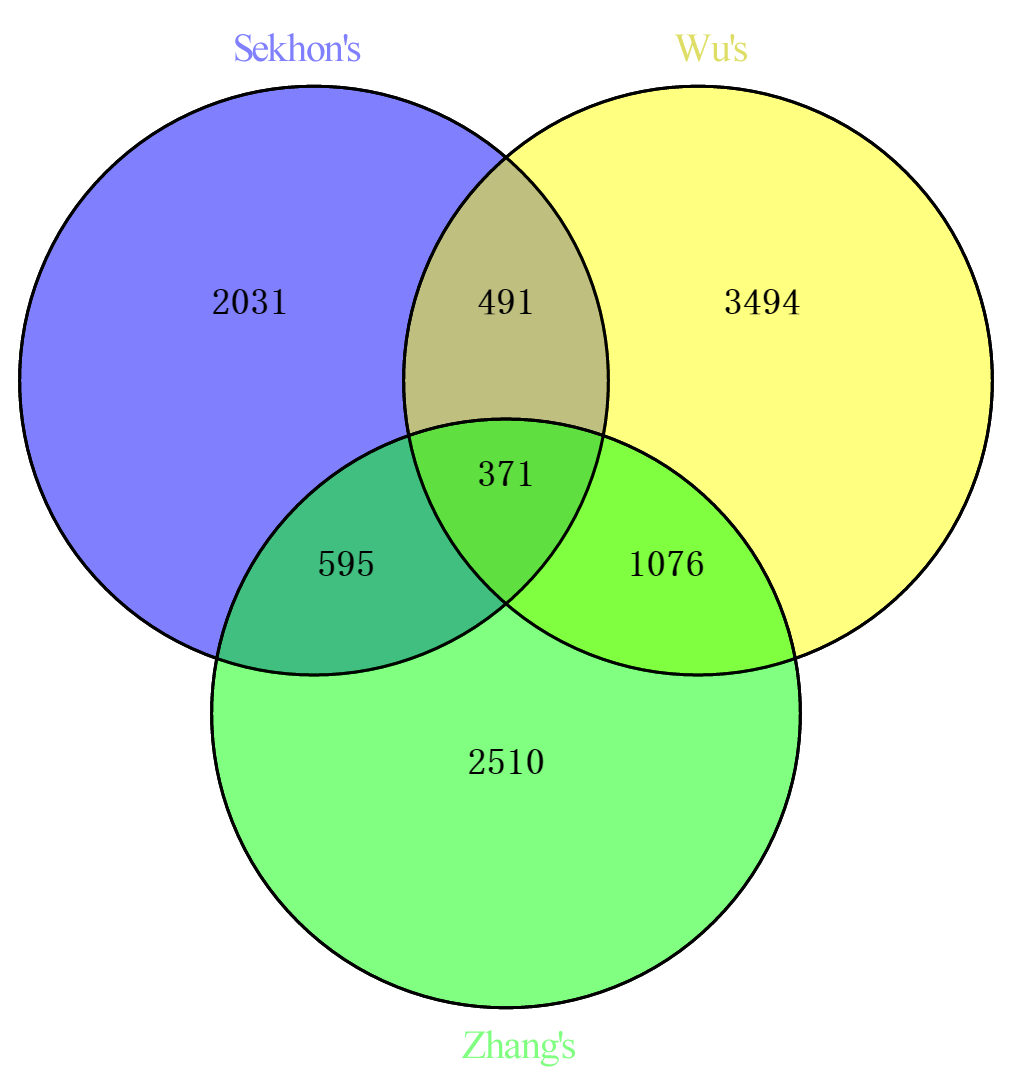

Supplement: S4 Fig — The green area represents the differentially expressed genes during natural leaf senescence by Zhang et al. (2014), the blue area represents the differentially expressed genes in B73 during early leaf senescence induced by prevent pollination by Sekhon’s et al. (2012), the yellow area represents the differentially expressed genes in this study. The areas shown in the diagram are not proportional to the number of genes in each group. (TIF) [file pone.0185838.s004.tif]

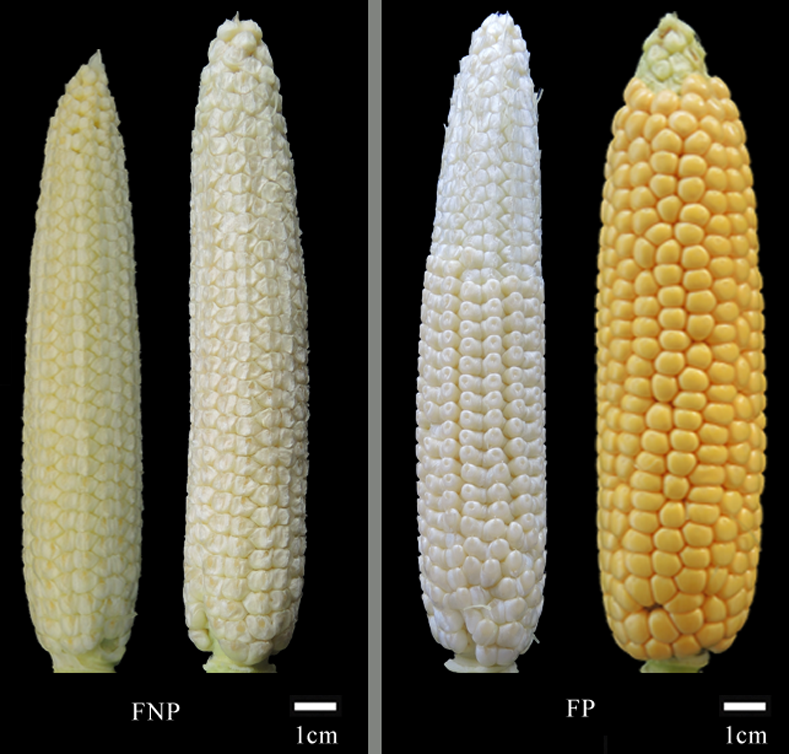

Supplement: S5 Fig — (TIF) [file pone.0185838.s005.tif]

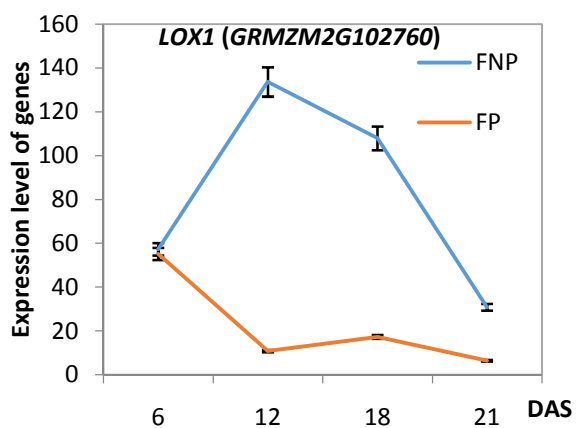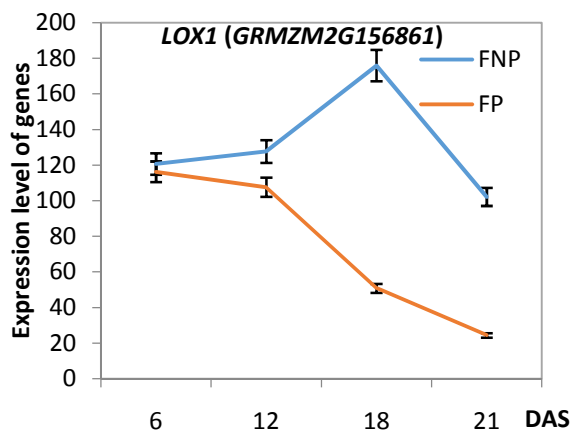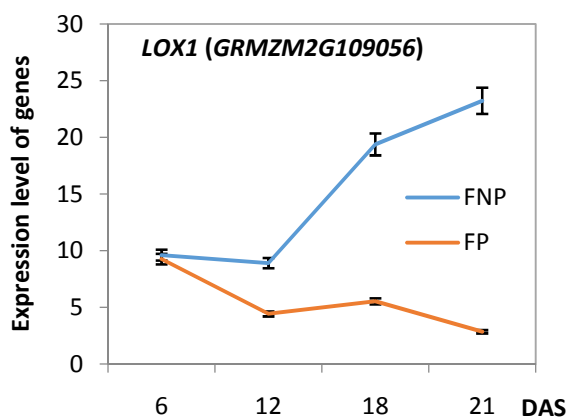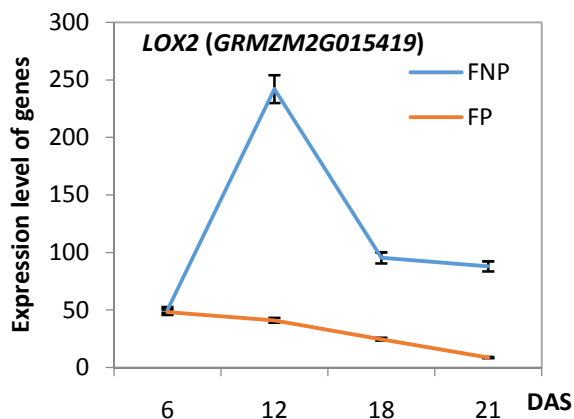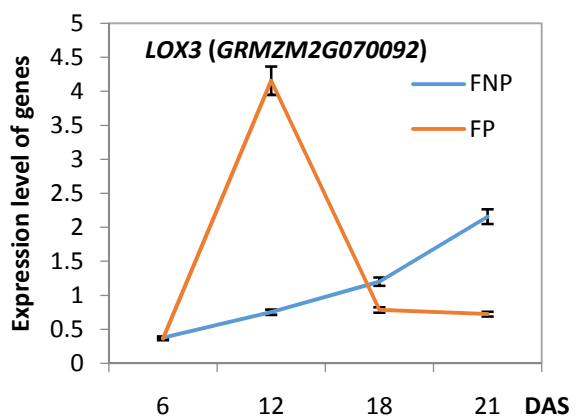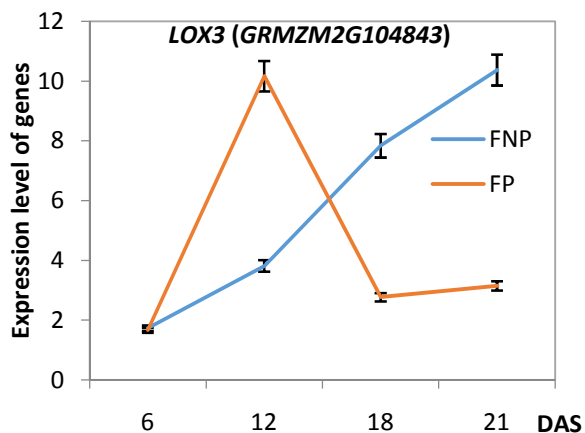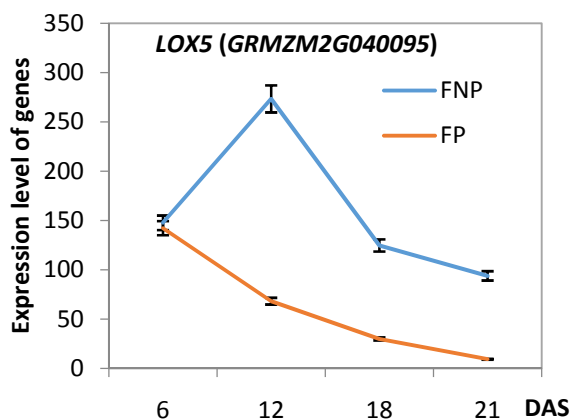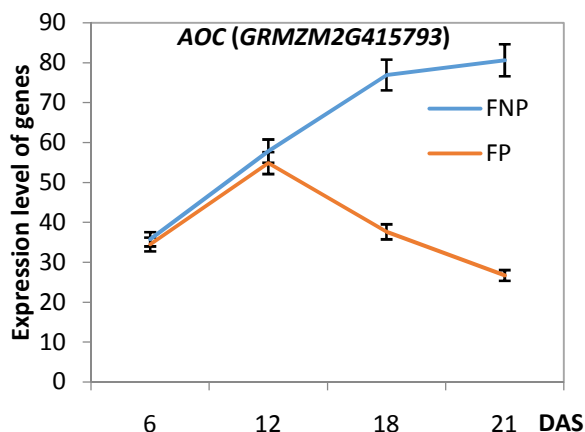

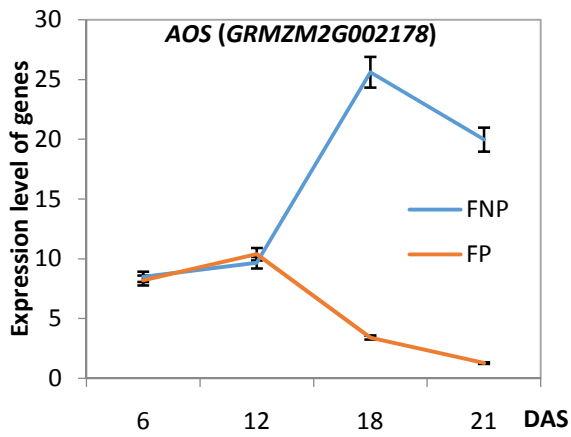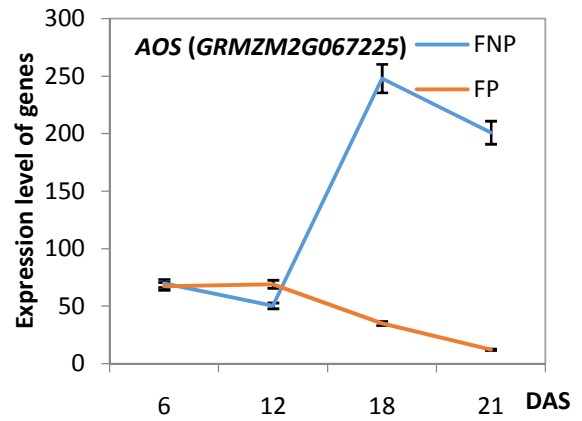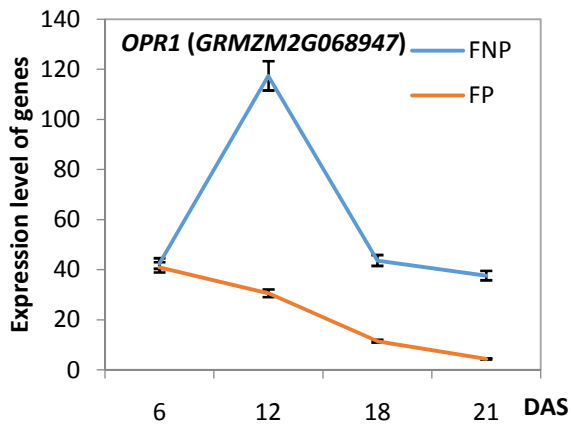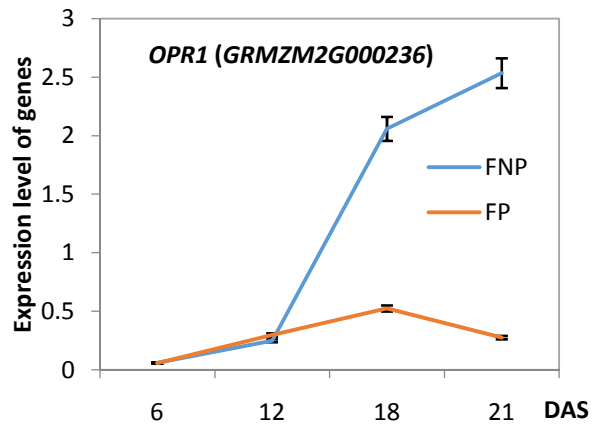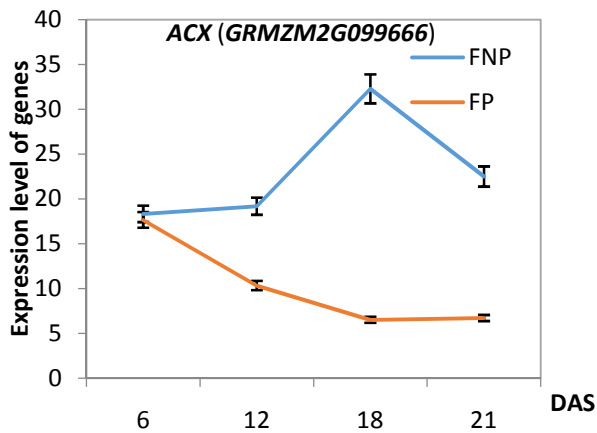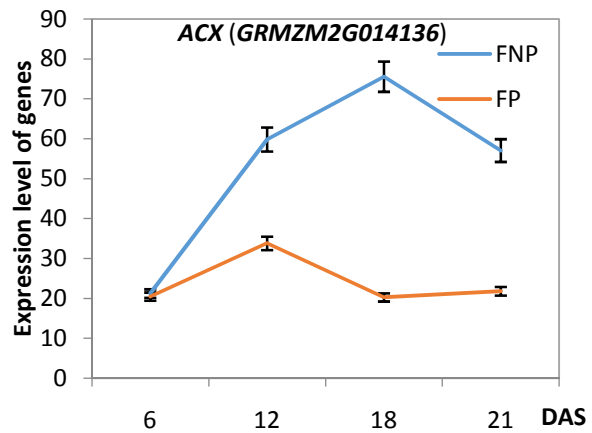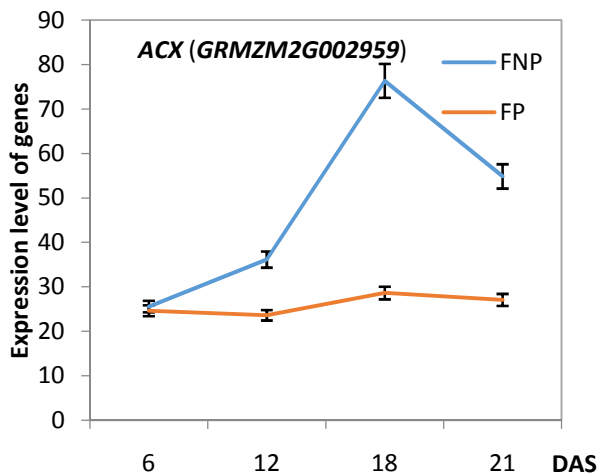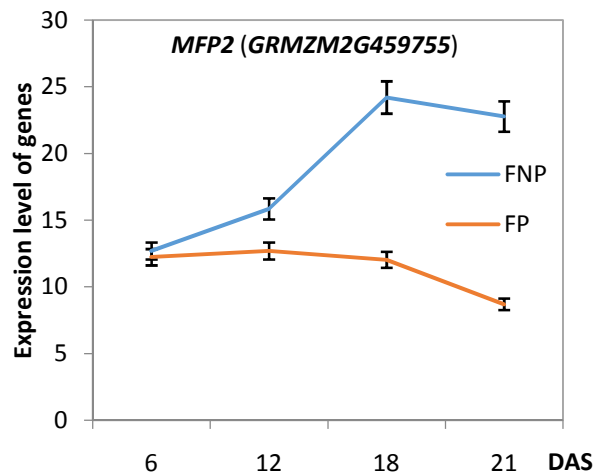

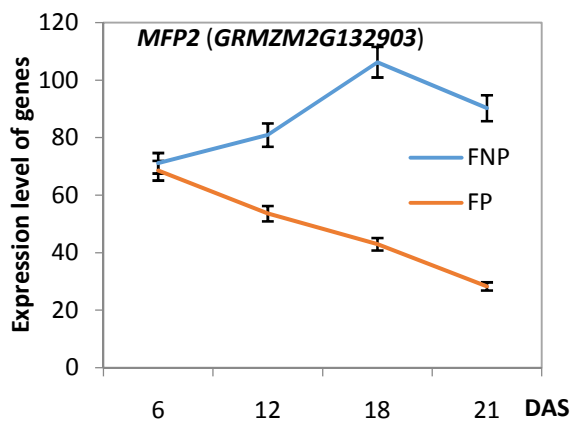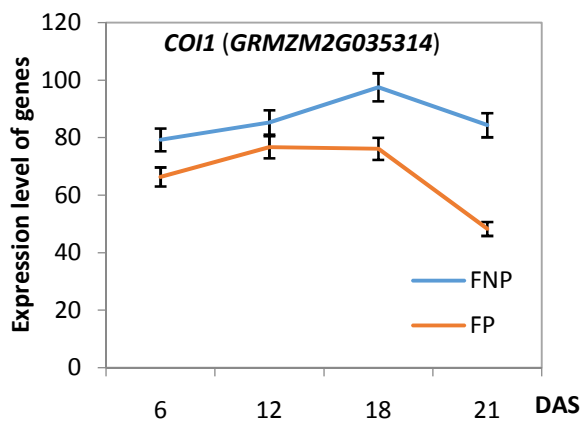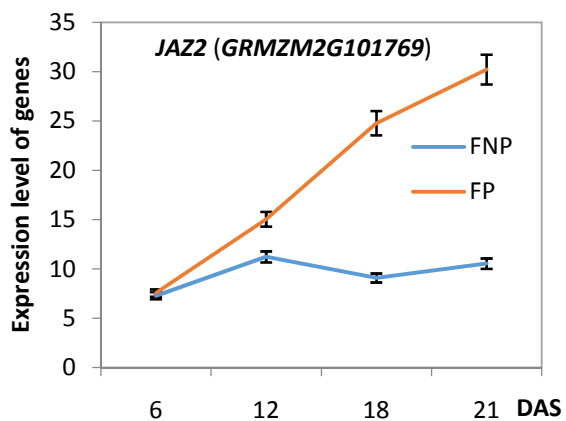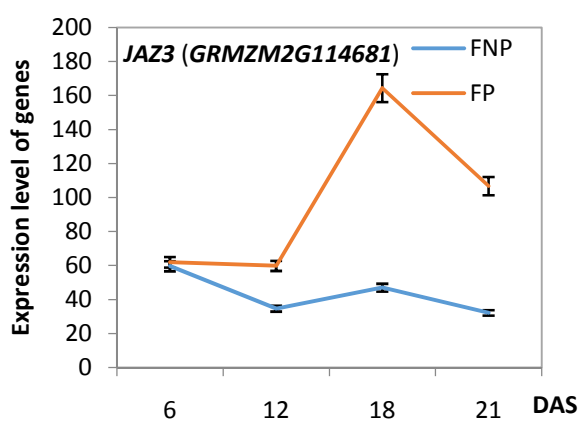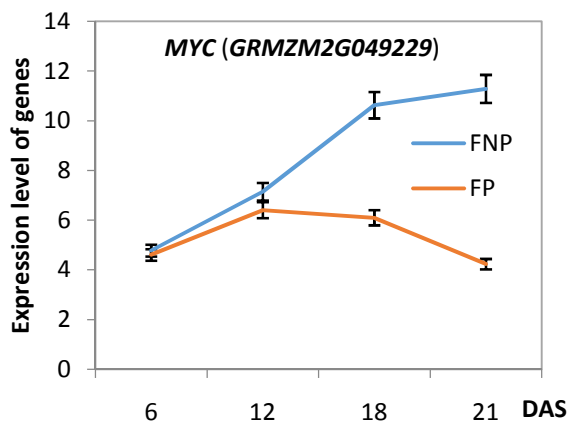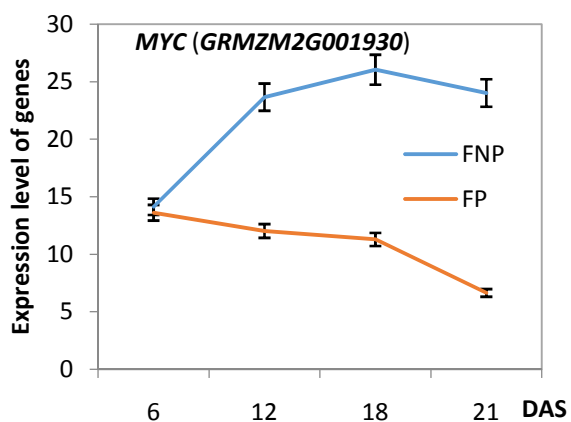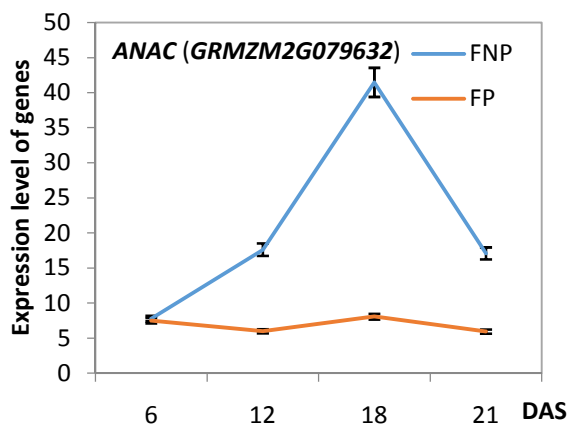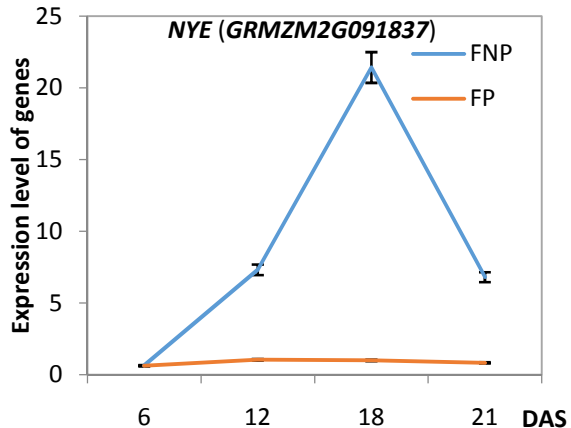

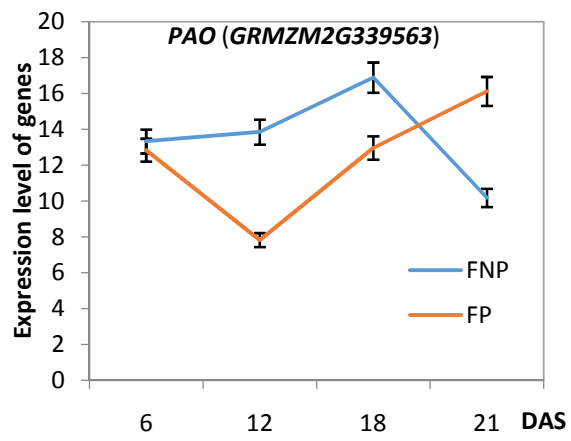

Supplement: S6 Fig — (PDF) [file pone.0185838.s006.pdf]

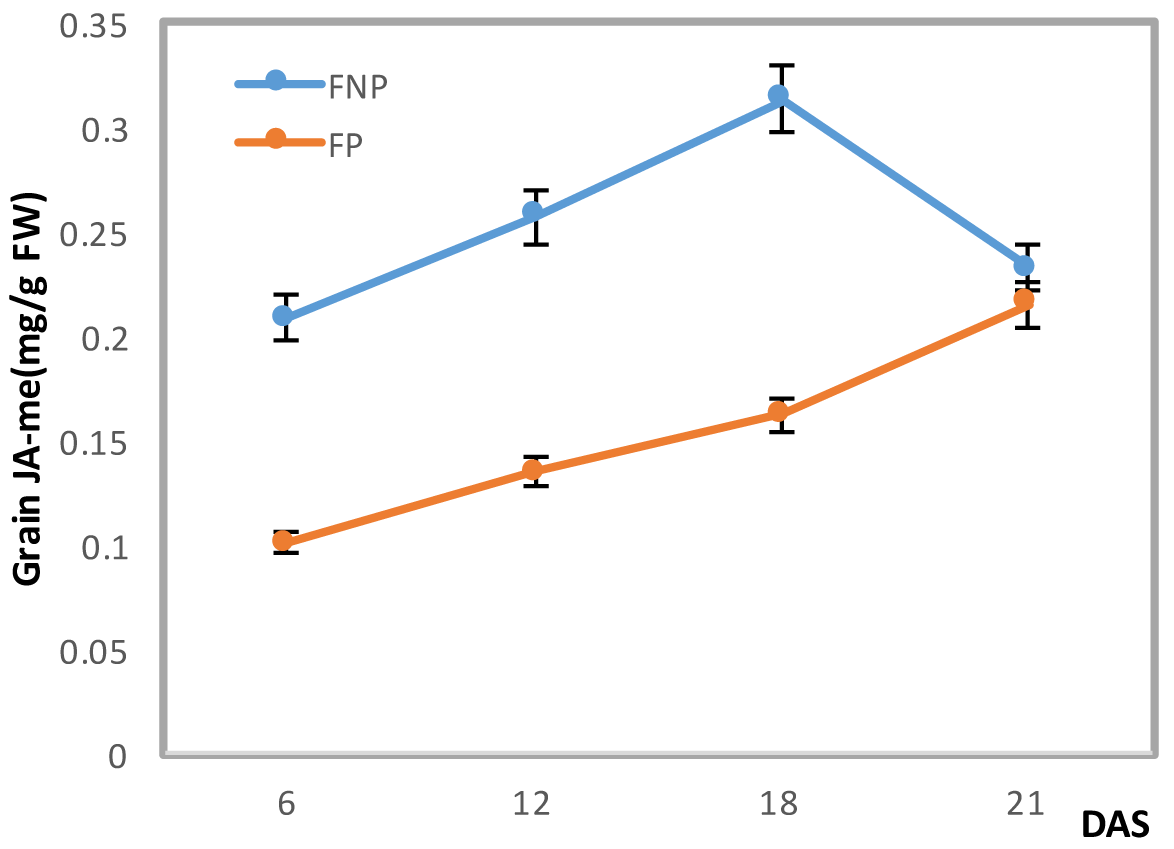

Supplement: S7 Fig — The bar represents the mean ± SE, n ≥ 8. (TIF) [file pone.0185838.s007.tif]
